# Two dominant forms of multisite similarity decline – Their origins and interpretation

**DOI:** 10.1002/ece3.9859

**Published:** 2023-03-08

**Authors:** David C. Deane, Cang Hui, Melodie McGeoch

**Affiliations:** ^1^ Department of Environment and Genetics, Research Centre for Future Landscapes La Trobe University Melbourne Victoria Australia; ^2^ Department of Mathematical Sciences, Centre for Invasion Biology Stellenbosch University Matieland South Africa; ^3^ Biodiversity Informatics Unit African Institute for Mathematical Sciences Cape Town South Africa

**Keywords:** conspecific spatial pattern, interspecific spatial pattern, macroecology, multisite turnover, simulation model, zeta diversity decline

## Abstract

The number of species shared by two or more sites is a fundamental measure of spatial variation in species composition. As more sites are included in the comparison of species composition, the average number of species shared across them declines, with a rate increasingly dependent on only the most widespread species. In over 80% of empirical communities, models of decline in shared species across multiple sites (multisite similarity decline) follow one of two distinct forms. An exponential form is assumed to reflect stochastic assembly and a power law form niche‐based sorting, yet these explanations are largely untested, and little is known of how the two forms arise in nature. Using simulations, we first show that the distribution of the most widespread species largely differentiates the two forms, with the power law increasingly favored where such species occupy more than ~75% of sites. We reasoned the less cosmopolitan distribution of widespread species within exponential communities would manifest as differences in community biodiversity properties, specifically more aggregated within‐species distributions, less even relative abundance distributions, and weaker between‐species spatial associations. We tested and largely confirmed these relationships using 80 empirical datasets, suggesting that the form of multisite similarity decline offers a basis to predict how landscape‐scale loss or gain of widespread species is reflected in different local‐scale community structures. Such understanding could, for example, be used to predict changes in local‐scale competitive interactions following shifts in widespread species' distributions. We propose multiple explanations for the origin of exponential decline, including high among‐site abiotic variation, sampling of highly specialized (narrow niche width) taxa, and strong dispersal limitation. We recommend these are evaluated as alternative hypotheses to stochastic assembly.

## Graphical abstract

Comparison of diversity patterns for the two dominant forms of multisite similarity decline (a) decline in similarity (b) species accumulation.

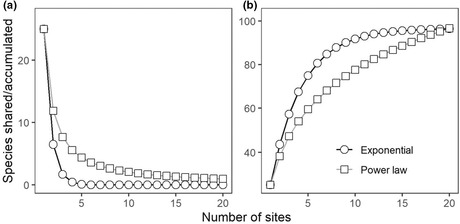

## 1 INTRODUCTION

**FIGURE 1:**
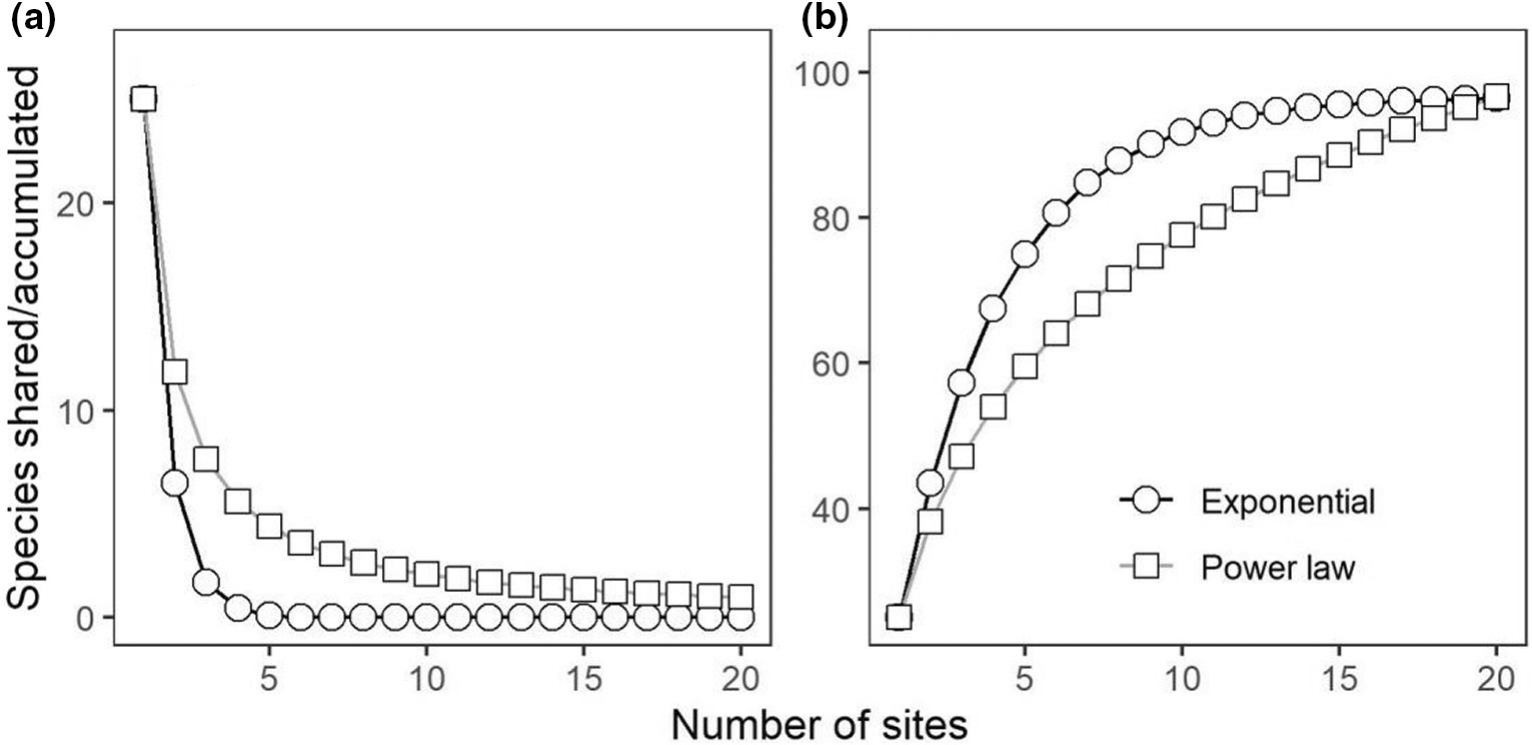
Differences in two patterns of diversity for theoretical exponential and power law communities with constant alpha and gamma diversity. (a) Multisite similarity decline (change in the average number of species shared across increasing numbers of sites) and (b) species accumulation (change in the average total number of species observed for increasing numbers of sites). Both communities were simulated with alpha diversity of 25 species and gamma diversity of 96 species, with curves calculated using formulae and parameter values in Hui and McGeoch (2014). For details on simulation, see Appendix S1.

Understanding how species are organized in space is a central question in theoretical and applied ecology. Given the important role of widespread species biodiversity patterns (especially species richness; Kreft et al., 2006; Lennon et al., 2004) and the differential environmental response of narrowly distributed versus widespread species (Latombe, Richardson, et al., 2018; Reeve et al., 2022; Wang et al., 2012), it is useful to explicitly ask how species of different occupancies influence species compositional change among sites in a community (Hui & McGeoch, 2014; McGeoch et al., 2019). A fundamental component of species compositional change over two or more sites is the number of species they share. The declining number of shared species with increasing number of sites compared succinctly describes how species of differing occupancy contribute to compositional change. This relationship of multisite similarity decline largely follows one of only two simple forms in empirical data (Figure 1), being either an exponential (26% best fit in tested empirical matrices) or power (57% best fit) function of the number of sites considered (Hui & McGeoch, 2014). No other commonly used occupancy model (Jenkins, 2011) is as well supported. This is of interest as it implies that – despite wide variation in empirical diversity patterns, community structures, and assembly mechanisms across different taxa and habitat types – two distinct patterns emerge. However, why most communities follow one or the other form of multisite similarity decline remains largely unexplored.

The two dominant models of multisite similarity decline do have different theoretical bases. Exponential decline can be viewed as a null model because it requires species to have the same probability of occurring at a given site (Hui & McGeoch, 2014). Equal probability of all species occupying a site has been used to infer that exponential‐form communities are dominated by stochastic assembly processes (Hui & McGeoch, 2014; Song et al., 2022). For example, more random dispersal might occur under high environmental flows. In contrast, whenever species differ in the probability they occupy a site as, for example, would be expected under niche theory, the exponential form will be rejected.

Because most studies adopt a dichotomous test between the exponential and power law forms (we will return to this point in Section 4), whenever the latter is found to be the better model it is typically interpreted as evidence of fine‐scale niche differentiation, dominating assembly processes (Hui & McGeoch, 2014). Empirically, as originally formulated, the form of decline has been found to differ within and between taxa and ecosystems, such as between flea and mite ectoparasites of small mammals, which, on the same hosts, follow exponential and power law forms of decline, respectively (Krasnov et al., 2020). It can also differ within a management context, for example, between endemic cichlid fish species in protected (power law) versus unprotected (exponential) areas of Lake Tanganyika (Britton et al., 2017). However, beyond invoking the broad theoretical distinction to explain these differences, there is no general expectation of the ecological conditions under which either form may be expected, limiting our ability to explain, predict, or interpret a finding of one or the other form.

One way to understand how species compositional patterns arise in nature is to take a bottom‐up approach, deconstructing the way individuals are distributed among species and how they are positioned with respect to other individuals. One such approach, sometimes called biodiversity macroecology (Leibold & Chase, 2018; McGill, 2010), has been widely used to simulate assemblages and to model and understand the origins of species–area relationships (Harte et al., 2008; He & Legendre, 2002), habitat subdivision (Deane et al., 2022; May et al., 2019), and variation in compositional similarity with sampling area and distance (Morlon et al., 2008; Plotkin & Muller‐Landau, 2002). Often validated using empirical data (e.g., Deane et al., 2022; Harte et al., 2008; Plotkin & Muller‐Landau, 2002), this approach offers a level of confidence in the causal, or at least practical, importance of these properties in producing observed compositional patterns. Because simulated communities can be sampled at any grain, this approach can also explicitly account for any effects of scale (Wiens, 1989), or equivalently, the number of sampled individuals (McGlinn et al., 2019), on the observed species composition.

Three properties of biodiversity in communities (hereafter community biodiversity properties; Table 1) are needed to specify the possible range in species compositional patterns (Chase et al., 2018; Keil et al., 2021; McGill, 2010; McGlinn et al., 2019): (1) the allocation of individuals to different species (species relative abundance distributions); (2) the placement of the individuals of each species with respect to others of their own species (conspecific spatial patterns); and (3) the placement of individuals with respect to the position of the individuals of other species (interspecific spatial patterns; Table 1). It is common in biodiversity theory to consider only the first two properties under the assumption that the individuals of each species are positioned at random with respect to those of other species (McGill, 2010). However, this assumption would be violated, or at least constrained, in the presence of strong species sorting along abiotic gradients as well as in some recognized empirical patterns of species co‐occurrence such as checkerboard pairs, where two species never co‐occur at the same site (Diamond, 1975). Matrix‐based simulations (e.g., Leibold & Mikkelson, 2002; Ulrich & Gotelli, 2010) can be used to understand the implications of such strong interspecific spatial patterns.

**TABLE 1:** Community biodiversity properties related to patterns of biodiversity variation and change with the metrics used to calculate these properties in this study and ecological contexts where different values of the property are predicted to arise (superscripts refer to sources in the footnote).

Community property	Metrics used in this study to quantify	Value	Ecological context observed as being associated or correlated with stated value
Relative abundance distribution (RAD)			
Measured as evenness [as drawn from the species abundance distribution (SAD)]	Probability of interspecific encounter (PIE)^1^	Low	Early successional stages^2^ High‐latitude or ‐altitude ecosystems^3^ Small fragments and islands^4^ Small sample size^5^ Polluted/disturbed systems^6^
		High	Climax community/low disturbance^7^ Fully censused continuous habitat^8^ Core species (omitting rare occasional species)^9^ Low‐latitude ecosystems^10^
Measured as rare species component of RAD	Proportion of in only one sample (single‐patch endemic species)^11^	High	Plant and invertebrate assemblages of small fragments^12^
Conspecific spatial pattern (CSP)			
Measured as direction (under vs. overdispersed vs. random) and strength	Morisita index. For individual species, or mean or other summary statistic for >1 species)^13^	Regular	Territorial taxa^14^ Negative density dependence (e.g., Janzen–Connell effect)^15^
		Random	Seasonally or cyclically disturbed sites^16^
		Aggregated	Dispersal limitation^17^ Heterogeneous abiotic environment^18^ Fragmented habitat^19^
Interspecific spatial pattern (ISP)			
Measured as spatial association (positive or negative)	Checkerboard score^20^ Pairwise covariance in abundance^21^	Low (more positive covariance)	Low environmental heterogeneity^22^ High dispersal/mass effects^23^
		High (more negative covariance)	Competitive exclusion^24^ Environmental filtering^25^ Dispersal limitation^26^ Priority effects^27^ Asynchronous disturbance (e.g., forest canopy gaps)^28^ Sampling multiple species pools^29^ Ecological drift^30^

*Note*: 1. Hurlbert (1971); 2, 7. Bazzaz (1975); 3, 10. Alroy (2015); 4. Chase et al. (2020), Deane et al. (2020), Gooriah et al. (2021); 5, 8. Preston (1962); 6, 7. Gray and Mirza (1979), Hill et al. (1995); 8, 17. Hubbell (2001); 9. Magurran and Henderson (2003); 11, 12. Simberloff and Gotelli (1984); 12. Bosc et al. (2019); 12, 16. Deane and He (2018); 13. Hurlbert (1990); 14. Järvinen (1992); 15. Janzen (1970); 16. Haila et al. (1993); 18. Getzin et al. (2008); 19. He and Hubbell (2013); 20. Stone and Roberts (1990); 21. Keil et al. (2021); 22, 23. Mouquet and Loreau (2003); 24. Diamond (1975); 25. Connor and Simberloff (1979); 26. Levin (1974); 27. Peart and Foin (1985); 28. Hubbell (1979); 26, 30. Ulrich (2004); 29. Nekola and McGill (2014).

Understanding the relationship between fine‐scale (e.g., within‐sample) assemblage structure (defined using community biodiversity properties) and compositional (among‐sample) patterns offers a powerful means to predict how changes either within or among samples will manifest in the other (He & Legendre, 2002; McGlinn et al., 2019; Plotkin & Muller‐Landau, 2002). Wherever ecological processes are associated with known (or hypothesized) changes in community biodiversity properties (examples in Table 1), one can predict the likely effects on composition. For example, low evenness in species relative abundance distributions is typical of early successional (Bazzaz, 1975) or fragmented (Chase et al., 2020) communities and, with all else held equal, will result in lower alpha and higher beta diversity compared to (presumably more even) late‐successional or continuous communities, respectively (He & Legendre, 2002; Plotkin & Muller‐Landau, 2002). Moreover, changes to community biodiversity properties can be used to disentangle changes, for example, in species–area relationships following invasion (Powell et al., 2013) or in understanding the mechanisms underpinning island species–area relationships (Gooriah et al., 2021). In this way, identifying any relationship between community biodiversity properties and the two dominant forms of multisite decline could offer a deeper understanding of their mechanistic origins and fine‐scale structure.

Here, we examine how the two dominant forms of multisite similarity decline arise, and whether they exhibit systematic differences in community biodiversity properties. Through simulation, we first reproduce the two forms by imposing different combinations and strengths of community biodiversity properties (Table 1). This allows us to ask which combinations are more likely to produce one or the other dominant form of multisite similarity decline. For example, is exponential decline more likely to be associated with more even or less even relative abundance distribution, stronger or weaker conspecific spatial patterns, and more positive or more negative interspecific spatial association? Having identified clear relationships through simulation, we then sought to validate the inferred relationships using empirical data. Finally, we discuss how the insights generated can be applied to deepen understanding of the origin of the observed form of decline and offer some suggestions on their potential application in hypothesis generation.

## 2 METHODS

### 2.1 Overview and terminology

Essentially, this study tests for the presence and strength of association between incidence‐based patterns of diversity and abundance‐based community properties. An expectation for how this might arise was first developed using simulation, and this was then tested using empirical data. Throughout, where the term “abundance” is used, this refers to relative numbers of individuals either within a site or within the community overall. Thus, species might be more or less abundant based on the relative numbers of individuals. When referring to species found in many sites, we use the term widespread species, and for those found in few sites, we use narrowly distributed. While abundant species are also likely to be more widespread, the two terms refer to distinct concepts throughout.

### 2.2 Quantifying compositional similarity across multiple sites

The minimum description of spatial community structure is an incidence (presence–absence) site‐by‐species matrix, where marginal totals quantify basic diversity properties of the community; row sums quantify the species richness of each site, and column sums reflect how widely, or narrowly, distributed each species is. Variation in species composition among multiple sites can be quantified using various measures of similarity, or its complement, dissimilarity, depending on the research question (Arita, 2017; Baselga et al., 2007; Diserud & Ødegaard, 2007; Hui & McGeoch, 2014). If the aim is to quantify overall heterogeneity, it is common to use a multisite dissimilarity metric (Baselga et al., 2007; Diserud & Ødegaard, 2007). These can be partitioned into contributions due to turnover and nestedness, allowing researchers to infer the relative importance of species replacement and species loss within that community (Baselga, 2010). Alternatively, if the aim is to understand how narrowly versus widespread species contribute to compositional change, one can use measures of similarity derived from species occupancies (Hui & McGeoch, 2014; McGeoch et al., 2019). In the latter case, rather than a single value capturing the overall heterogeneity in the community, compositional similarity can be depicted as a function of any number of sites combined (as distinct from increasing distance between them; Nekola & White, 1999). The shape of the curve is monotonically declining; thus, we refer to this relationship as multisite similarity decline.

### 2.3 Modeling the form of multisite similarity decline

To calculate multisite similarity decline, one starts by quantifying the number of species shared by pairs of sites, triplets of sites, and so on, potentially up to the number of species shared across all sites. These quantities are known as zeta diversity and the order of zeta refers to the number of sites included in the combination (Hui & McGeoch, 2014). In the same way that a species accumulation curve captures information about the average *increase* in species number as sites are combined, an analogous curve can be built that quantifies the average *decline* in the number of species shared (Figure 1). As with species accumulation, to compare among different communities, it is convenient to model the form of decline. If zeta diversity $\zeta_{i}$ is the number of species shared on average among *i* sites in a dataset (*i* designating the order), then an exponential model of decline takes the form: $\zeta_{i}=a\cdot \exp\left(-b\cdot i\right)$ and power decline $\zeta_{i}=c\cdot i^{-d}$, where *a*, *b*, *c*, and *d* are model parameters (Hui & McGeoch, 2014). Notably, a third model combining the power and exponential forms, the power–exponential function $\zeta_{i}=a\cdot i^{-b}\cdot \exp\left(-c\cdot i\right)$, provides more flexibility in modeling multisite decline (e.g., Latombe et al., 2019; Lazarina et al., 2019). This model has significant potential for exploring the ecological variability within and across communities; however, our intention here is to better understand the null model (i.e., exponential decline) and established alternative power law forms.

Intuitively, one might expect multisite similarity decline and species accumulation curves to be related, and indeed, species accumulation curves can be expressed as a function of the number of species shared for different numbers of sites (Hui & McGeoch, 2014). Additionally, decline in shared species for an increasing number of sites exclusively depends on the contribution of more widespread species. By simulating multisite similarity decline and species accumulation curves for exponential and power law form communities with the same alpha (i.e., mean of row sums [richness]) and gamma (i.e., number of columns [total species]) diversity under identical sampling effort (number of rows [sites]), we can develop some appreciation for what each represents. It is clear from this exercise that an exponential form declines more rapidly over all orders, meaning fewer species are shared (Figure 1a). This has the corollary that a greater proportion of new species are encountered, resulting in faster saturation of the accumulation curve relative to power law form communities (Figure 1b, Appendix S1).

### 2.4 Simulations

#### 2.4.1 Abundance‐based null models

We first used a spatially implicit abundance‐based null model for the expected number of shared species in samples from an assemblage (Deane et al., 2022). The null model generates a set of randomly (independently) positioned samples in a continuous landscape without strong directional abiotic gradients. This model enabled us to test the effect on the form of multisite similarity decline when independently varying the number of individuals (modeled using varying sampling grain under a constant individual density assumption), species' relative abundances, and the amount of aggregation in their conspecific spatial patterns. Under the common assumption in biodiversity theory that the individuals of each species are positioned independently of the position of other species (McGill, 2010), these three factors together determine patterns of diversity at any scale (Chase et al., 2018). The intention here was to identify their individual influence on the form of multisite decline.

Assuming the statistical distribution of individuals of all species is described by a finite negative binomial distribution (Zillio & He, 2010), the null model takes the form (Deane et al., 2022):

(1) $$\zeta_{m|\alpha }=\sum_{i=1}^{S}{\left[1-\frac{\Gamma \left(N_{i}+\left(k_{i}/\alpha \right)-k_{i}\right)\Gamma \left(k_{i}/\alpha \right)}{\Gamma \left(N_{i}+\left(k_{i}/\alpha \right)\right)\Gamma \left(\left(k_{i}/\alpha \right)-k_{i}\right)}\right]}^{m}$$

where *α* is the proportion of the total sampling extent *A* contained in a single sample of area (i.e., sampling grain) *a*; *N*_*i*_ is the total abundance of species *i* in total area *A*; *k*_*i*_ is a parameter controlling conspecific aggregation (here ranging from 0.2 to 10 reflecting highly aggregated to nearly random placement of individuals); *m* is the number of samples (zeta order); and Γ(*n*) = (*n* − 1)! is the gamma function. For a given *α* (= *a*/*A*), individuals of each species are assumed to follow density‐dependent conspecific aggregation $k_{i}=kN_{i}\alpha$, where k is a scaling factor used to model scale dependency in the strength of conspecific spatial aggregation of the community (e.g., He & Legendre, 2002; Plotkin & Muller‐Landau, 2002; Zillio & He, 2010).

Simulations varied the sampling grain (*a*), conspecific aggregation (achieved by varying scaling factor k), and species abundance distribution (*N*_*i*_) to calculate zeta diversity. Observed multisite similarity decline was then fitted to the two different parametric forms and the most likely form for each set of conditions identified (see Post‐processing of simulations). All abundance‐based simulations were based on the 50‐ha Barro Colorado Island stem‐mapped forest plot (Condit et al., 2012) and the 1995 census (301 species ~211,000 individuals). We assumed the level of conspecific aggregation scaled with the observed data (i.e., with parameter *k* = 0.94 at 400 m^2^; Deane et al., 2022) and varied k with sampling grain accordingly, over the range 25 m^2^ to 1 ha. To assess the effects of species' relative abundance, we used three common distributions, which vary in their evenness: log series, lognormal, and broken stick (listed in order of increasing evenness).

From the results of the abundance‐based simulations (see Section 3), we selected a sampling grain of 100 m^2^ to differentiate any effects of conspecific spatial pattern. We note this is somewhat unrealistic, being well below the typical minimum quadrat size used in forest ecosystems of 400 m^2^ (Rodríguez‐Hernández et al., 2021). However, use of larger grain sizes did not produce exponential decline for any relative abundance distribution or conspecific spatial pattern. At this constant sampling grain, we altered the intensity of aggregation in conspecific spatial patterns by varying the scaling parameter. The range of values corresponded to assemblages following a gradient from highly aggregated conspecific spatial patterns (equivalent value of *k* = 0.1) toward random placement (*k* = 10). We repeated this for each of the three abundance distributions and each scenario we fit the two forms of decline (see Post‐processing of simulations).

#### 2.4.2 Matrix‐based spatially semi‐explicit simulations

As discussed above, the abundance‐based null model assumes the individuals for each species can be placed independently (i.e., without regard for the location of individuals of other species). While a common assumption in biodiversity models (McGill, 2010), this does not allow one to explicitly test the effects of different interspecific spatial associations (e.g., species sorting along abiotic gradients or competitive exclusion) on biodiversity. To test for the role of interspecific spatial patterns, we, therefore, used matrix‐based, spatially semi‐explicit simulations based on both realistic and idealized community structures (such as checkerboard and nested community structures; Presley et al., 2010). Spatially semi‐explicit (Hui et al., 2010) means that the relative positions of species to each other are known while their absolute coordinates are irrelevant and thus unspecified. We simulated presence–absence matrices following different assumptions about interspecific spatial associations at the level of the site and tested the effect of these on the observed form of multisite similarity decline. Matrix dimensions used were consistent with prior studies (e.g., 20–50 sites × 20–50 species; Leibold & Mikkelson, 2002; Ulrich & Gotelli, 2010); however, the outcomes for multisite similarity decline were qualitatively robust and results presented are based on a 20 × 20 (sites by species) matrix.

Rather than pursuing an exhaustive list of possible structures, the aim here was to test the most likely form of decline as the interspecific association among species became more negative (i.e., greater segregation among species). As the abundance‐based simulations demonstrated a strong influence of sampling grain and therefore matrix fill (Section 3), all matrix‐based simulations were intentionally created to have a high proportion of filled cells (matrix fill ~0.5). This ensured any influence on the form of decline was due to interspecific spatial associations and not matrix fill. We note this value of fill is high for empirical data, for example, being exceeded in only ~20% of the 290 datasets compiled by Atmar and Patterson (1995), which have a mean ± SD of 0.24 ± 0.13 (Hui & McGeoch, 2014). We also note that matrix fill (being the sum of species richness of all sites [equivalently, the sum of occupancies of all species] divided by the product of the number of sites and total number of species) is the inverse of Whittaker's (Whittaker, 1960) multiplicative beta diversity partition (Arita, 2017) and therefore has a biological interpretation as the ratio of mean sample richness (α‐diversity) and total observed species (an estimate of γ‐diversity). It is also equivalent to connectance in bipartite network and co‐occurrence theory (Gotelli & Graves, 1996; Henriksen et al., 2022).

Five different matrix simulations are presented in the main text (see Appendix S2 for additional structures and background on simulations). Three represented an idealized structure, simulating deterministic controls on species presence within sites and fully cohesive occupancies (i.e., with no gaps in each species' range; Leibold & Mikkelson, 2002): (1) fully nested subsets (“nested”), for example, resulting from selective extinction of species from habitats ordered in decreasing size; (2) equal niche‐width abiotic gradients (i.e., “evenly spaced gradients”; Leibold & Mikkelson, 2002) simulating species sorting according to unique niche positions along an abiotic gradient; and (3) a Clementsian gradient comprising two separate communities (e.g., resulting from sampling across a sharp range boundary), with two species common to both communities (“turnover compartmented”; Ulrich & Gotelli, 2013). The other two matrices simulated more biologically realistic communities using an individual‐based model similar to Kraft et al. (2011). Here, the total number of individuals in each site, drawn from a Poisson distribution and distributed among species according to a vector of sampling probabilities, varied to simulate different interspecific associations. Simulated abundances were converted to presence or absence for further analysis. In the first biologically realistic matrix (“continuous community”), individuals were assigned to species according to a log‐normal occupancy probability, representing samples obtained from a continuous landscape. In the second realistic matrix (“ecotone”), sampling from two communities separated by a transitional ecotone was simulated using log‐normal occupancy probabilities oriented in reverse directions for species 1–9 and 12–20. Species 10 and 11 were assumed to represent species of broad ecological tolerance, and their occupancies were sampled from a binomial distribution with a probability of 0.9.

A purely random matrix (i.e., one where every site had an equal probability of sampling every species) will have an exponential form of decline. Having simulated matrices with an imposed structure, we then asked two questions: what is the form of decline in the unperturbed matrix structure, and for structures favoring a power law form, how much perturbation in the structure is required to change the best‐supported form from power to exponential? The perturbation algorithm proceeded at each iteration by selecting one row (site) of the matrix randomly. Within each selected site, a swap was performed between a presence and an absence of species. Swaps were constrained to species with at least two occupancies in the matrix to maintain total species number. The number of species at each site and total matrix fill were thereby preserved, but the sites occupied by species (other than species at a single site) were free to vary. With each of 100 iterations of the randomized perturbation algorithm, support for the two forms of multisite decline was calculated (see the following section). Based on the results of the simulations, we present the change in relative support for the exponential form as a function of the corresponding *C*‐score (quantifying interspecific association as the number of checkerboard pairs of species; Stone & Roberts, 1990). We repeated this perturbation procedure 100 times for each starting structure and present the mean result across all simulations, where change in support for the two models of decline as a function of the *C*‐score is illustrated using a scatterplot (see next section).

#### 2.4.3 Assessing support for exponential versus power law decline

In all analyses of simulations (and empirical data below), we fit the exponential and power law forms using the linearized form of the exponential and power law models (Latombe, McGeoch, et al., 2018). For each simulated community (and randomization permutation), relative support for the two functional forms of decline was determined using the weight of support calculated from Akaike's information criterion (*w*AIC). To calculate *w*AIC, the relative likelihood of each model is calculated as $\exp\left(-0.5\cdot ∆\mathrm{AIC}\right)$, where ∆AIC is the difference in AIC from the top‐ranked model. The Akaike weight for a model is this value divided by the sum of these values across all models, thus giving the relative likelihood of each model in the candidate set. A value of 1 for *w*AIC indicates no support for the alternative model (which in our case is always the power law form) and a value of 0.5 represents equal support for both models. Our main interest in simulations was in identifying which form of decline was favored for a given combination of community biodiversity properties or imposed matrix structure, and in the latter case, how disturbed (i.e., randomized) these structures would need to become to follow this (exponential) form.

### 2.5 Empirical tests

#### 2.5.1 Empirical communities

Empirical data were obtained from the meta‐Community Ecology: Species, Traits, Environment and Space (CESTES) database (Jeliazkov et al., 2020), which includes community data from 80 datasets collated from 57 published studies. The data include 13 different taxonomic groups (predominantly plants, insects, and vertebrates) collected from around the globe over a wide range of sampling extents and across the terrestrial, marine, and freshwater realms (Jeliazkov et al., 2020). To allow us to calculate the community biodiversity properties of interest (Table 1), we selected only those datasets from within this database with a quantitative measure of species performance at each site. Data recorded as number of individuals (*n* = 46), and abundance scores (*n* = 9) were used directly, while percentage cover (*n* = 6) or other metrics (*n* = 8; including log‐transformed, relative, or mean abundance) were back‐transformed where appropriate and then rounded to integer values (see provided Metadata, Supporting Information). This yielded 69 datasets quantifying or estimating relative abundance of each species in every sample and these values were treated as the number of individuals for quantifying community biodiversity properties. Six of these included either control and treatment plots (e.g., grazed and ungrazed), or featured multi‐year replicates. We, therefore, subdivided these datasets to ensure that we tested the form of multisite decline within individual study systems. In splitting these datasets, we coincidentally again obtained a total of 80 datasets (hereafter “communities”) from which we could relate the observed form of decline to community biodiversity properties.

#### 2.5.2 Statistical analyses

Because of variation among datasets in the number of sites and species, a decision was required on the maximum order of zeta to fit the model of decline for comparative purposes. While in practice this will depend on the questions considered (McGeoch et al., 2019), this is a non‐trivial decision because the form of multisite similarity decline changes depending on the maximum order of zeta considered (e.g., Latombe et al., 2019; see Appendix S3). Sensitivity testing of the empirical data showed that as the number of orders used increases, the exponential form is increasingly favored despite neither model fitting the data well (Appendix S3: Figure S1). This cautions against fitting to all orders indiscriminately but does not provide a recommended number of orders to calculate. One solution that has previously been proposed is to fit only to orders of zeta where at least one species is shared (Latombe et al., 2019; McGeoch et al., 2019). However, if the most widespread species occur in less than ~70% of sites, zeta diversity will fall below 1 after combining only two or three sites (Appendix S3: Figure S2). Zeta diversity will take a value of 0 if fit to more sites than are occupied by the most widespread species, so this represents the theoretical maximum order to use. However, we recommend at least five sites are used to ensure the contribution of widespread species is adequately captured. If we denote the proportion of sites occupied by the most widespread species as *p*_1_, if *p*_1_ = 1 (i.e., the most widespread species is found at every site), then multisite decline can be modeled using all sites; if *p*_1_ < 1, then the maximum number of orders should be the number of sites occupied by the most widespread species or $\ln\left(0.05\right)/\ln\left(p_{1}\right),$ whichever value is smaller (Appendix S3). However, it is also critically important to inspect the fit of the model.

To avoid issues associated with identifying the best‐supported form among datasets, here we used the first 10 orders to assign communities to either exponential or power law form of decline. Using fewer sites did not adequately differentiate between the forms (>10% of datasets fell within 2 AIC of the higher ranked model, indicating comparable support for both forms), and using a higher number of orders was limited by the available range of sites in the data. For example, using 20 orders would have precluded 25% of datasets. We manually inspected each fit to ensure the better‐supported form provided a reliable qualitative description of the data. Note that the classification of the communities was unchanged if all orders where zeta diversity remained above 1 were used, provided at least 10 orders were used to fit the models. When the minimum number of orders was reduced to five, three communities were reclassified (all from exponential to power law) but the results for the community biodiversity properties remained qualitatively the same (Appendix S4: Figure S1).

Having classified the 80 empirical communities as either exponential or power law, we tested for differences in community biodiversity properties (Table 1) between the two forms. To quantify the evenness of the distribution of species abundances, we used Hurlbert's (Hurlbert, 1971) probability of interspecific encounter (PIE), using the R package “mobr” (McGlinn et al., 2021). This value represents the probability that two individuals selected at random from an assemblage are of the same species and is advocated as a measure of evenness independent of sample size (Chase et al., 2019). The mean value of PIE was calculated for both among sites and for the pooled abundances across all sites. This then provides an indication of whether the form of decline reflects differences in evenness between local samples from the regional species pool, or from the underlying distribution of abundance across the landscape.

For interspecific and conspecific spatial patterns, our main interest was in comparing community biodiversity properties among the most widespread species in each dataset because of their influence on the observed form of decline (Appendix S4). Given the large variation in total species numbers between datasets (median total species [95% CI] = 50 [10, 158]), this required a decision on how best to select a subset of widespread species for comparison. We chose to use the four most abundant species to provide a constant number of comparisons for interspecific and conspecific spatial patterns. To test sensitivity in conclusions to this result, we also calculated the values using all species in the top 25th abundance percentile. Results were qualitatively the same for all community biodiversity properties (Appendix S4) and we believe the results to be robust to this decision.

To further control for differences in matrix dimensions in the communities, we calculated a standardized effect size in addition to the raw metrics (Ulrich & Gotelli, 2013), $\mathrm{SES}=\left(\mathrm{Obs}-\text{mean}(\mathrm{Exp}\right))/\mathrm{SD}\left(\mathrm{Exp}\right)$, where the mean and standard deviation of the expected (Exp) values were calculated from 999 matrices created using an appropriate null model.

To compare conspecific spatial patterns, we used the mean of the Morisita index of aggregation (Crist et al., 2003), which is interpreted as the probability that two individuals of the same species selected at random are found in the same sample (Hurlbert, 1990). This index is recommended because it scales linearly with grain, is intuitive to interpret, and is comparatively independent of abundance (Hui et al., 2010). For the Morisita index SES, the null model algorithm simulated random allocation of individuals among samples while retaining row and column marginal totals (function “r2dtable” in R).

To quantify interspecific spatial patterns, the mean value of Spearman's rank correlation between untransformed abundance vectors was used. This has the advantage over other metrics of segregation (e.g., the *C*‐score) in measuring the strength of both positive and negative interspecific association and in requiring no data transformation to meet distributional assumptions (Keil et al., 2021). Again 999 simulated communities were used to generate a reference distribution but here the null model was a non‐sequential, individual‐based quantitative shuffle algorithm (“c0_both”) using the R package “vegan” (Oksanen et al., 2020). This algorithm retains the total individuals and number of occupancies for each species, but individuals are shuffled among sites, removing any correlation in abundance while preserving the number of occupancies.

We also calculated the incidence‐based measure of interspecific association used in simulations, the *C*‐score (Stone & Roberts, 1990), which quantifies the number of observed checkerboard pairs relative to the maximum possible number given the observed occupancies. To calculate a reference distribution and SES for the *C*‐score, we used a non‐sequential binary null model algorithm (“curveball”) that preserves row and column marginal totals (Strona et al., 2014), again using the R package “vegan” (Oksanen et al., 2020).

We present a comparison of the values of each of the community biodiversity properties following the two forms of decline in the main text, testing for statistical differences in the distribution of community biodiversity properties for both raw and SES metrics using logistic regression where exponential communities represented a success (i.e., a value of 1), with a random term included to control for non‐independence of multiple datasets from individual studies. We also tested for the importance of the number of sites and total number of species along with ecological correlates provided in the CESTES database: study extent (km^2^), realm (marine, freshwater, and terrestrial), level of anthropogenic impact, and kingdom (producers and animals). All correlates were tested for influence on the observed form of decline using the same approach as for community biodiversity properties. We include other supporting results, including pairwise scatterplots of the relationships among the community biodiversity properties in the Supporting Information (Appendix S4). All simulations and analyses were done with R (Version 4.1.2).

## 3 RESULTS

### 3.1 Abundance‐based null models

**FIGURE 2:**
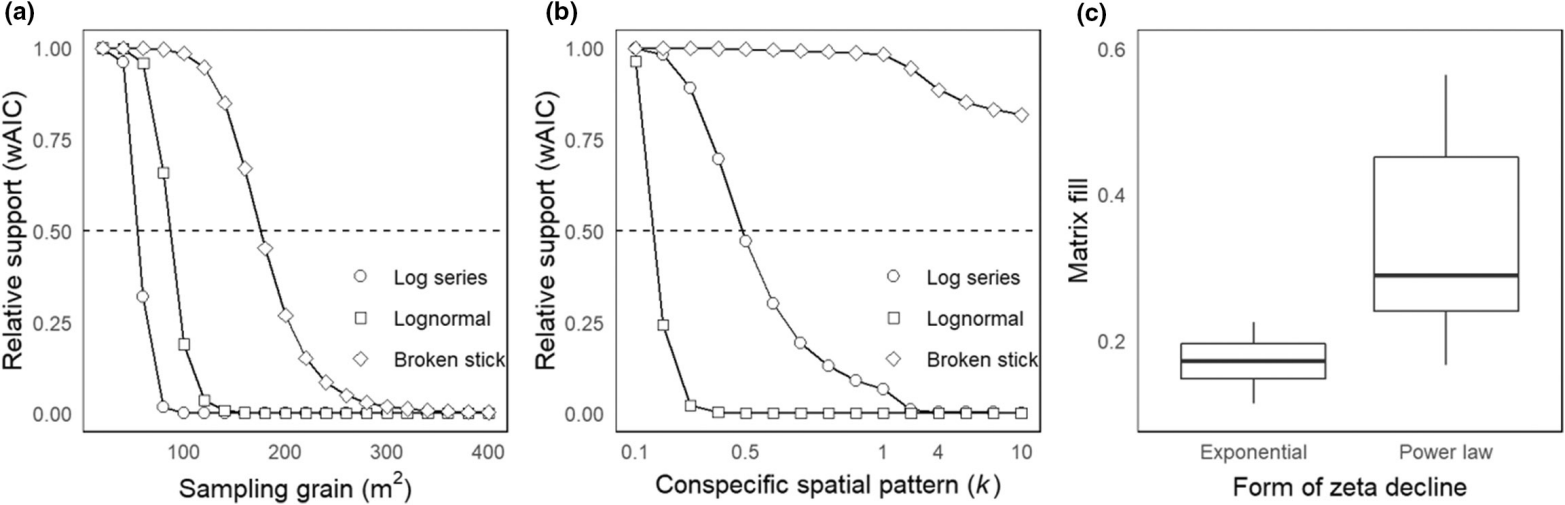
Change in the relative support for the exponential form of multisite similarity decline in simulated communities. A value of 1 indicates 100% support for the exponential form under scenarios of increasingly even distributions of species abundance (log series < lognormal < broken stick), moderated by (a) sampling grain, and (b) conspecific spatial pattern at 100 m^2^ sampling grain quantified using the *k* parameter of the negative binomial distribution. (c) Differences in matrix fill (calculated as mean alpha/gamma diversity) across scenarios in (a) and (b) that favor exponential versus power law decline. In (a) and (b), the *y*‐axis shows relative support for the two forms using the Akaike weight (*w*AIC). In (c), boxes show the median (dark line) and interquartile range, while vertical lines are 1.5 times the interquartile range of the matrix fill metric (low metric values reflect lower matrix fill). All simulations were based on sampling a 50‐ha hectare extent, with total species (=300) and individuals (=211,000) distributed according to the three relative abundance distributions. Note that using larger sampling grains than used for (a) does not alter relative support (Appendix S2: Figure S1).

Support for the power decline, that is, the rejection of exponential form, was more likely with: 1. an increase in grain size, 2. decline to less even relative abundance, and 3. weaker conspecific aggregation. Dependence on sampling grain is evident in the results of sampling from a simulated, continuous 50‐ha forest landscape (Figure 2a). Irrespective of the shape of the relative abundance distribution or conspecific spatial pattern for the assemblage, these simulations found no support for an exponential form once sampling grain exceeded ~400 m^2^ (Figure 2; Appendix S2: Figure S1). At a finer sampling grain (here illustrated at 100 m^2^), increasingly even relative abundance distributions increased the probability of observing exponential decline (log series < lognormal < broken stick). Stronger conspecific aggregation (reducing *k* at 100 m^2^ grain; more statistical overdispersion of individuals within species) also increased the probability of an exponential form (Figure 2b). Notably, although the exponential form is theoretically associated with stochastic assembly, as the simulation approaches random placement of individuals (i.e., with *k* = 10 or above; Figure 2b), support for the exponential form declines, that is, stochastic assembly is conceptually different from random placement of individuals. This dependence of the more likely form of decline on sampling grain, typical of the pervasive influence of spatial scale on biodiversity patterns (Azaele et al., 2015), is also evident in the relative matrix fill of simulations following the two forms, where exponential decline is associated with markedly lower fill (Figure 2c).

### 3.2 Matrix‐based simulations

**FIGURE 3:**
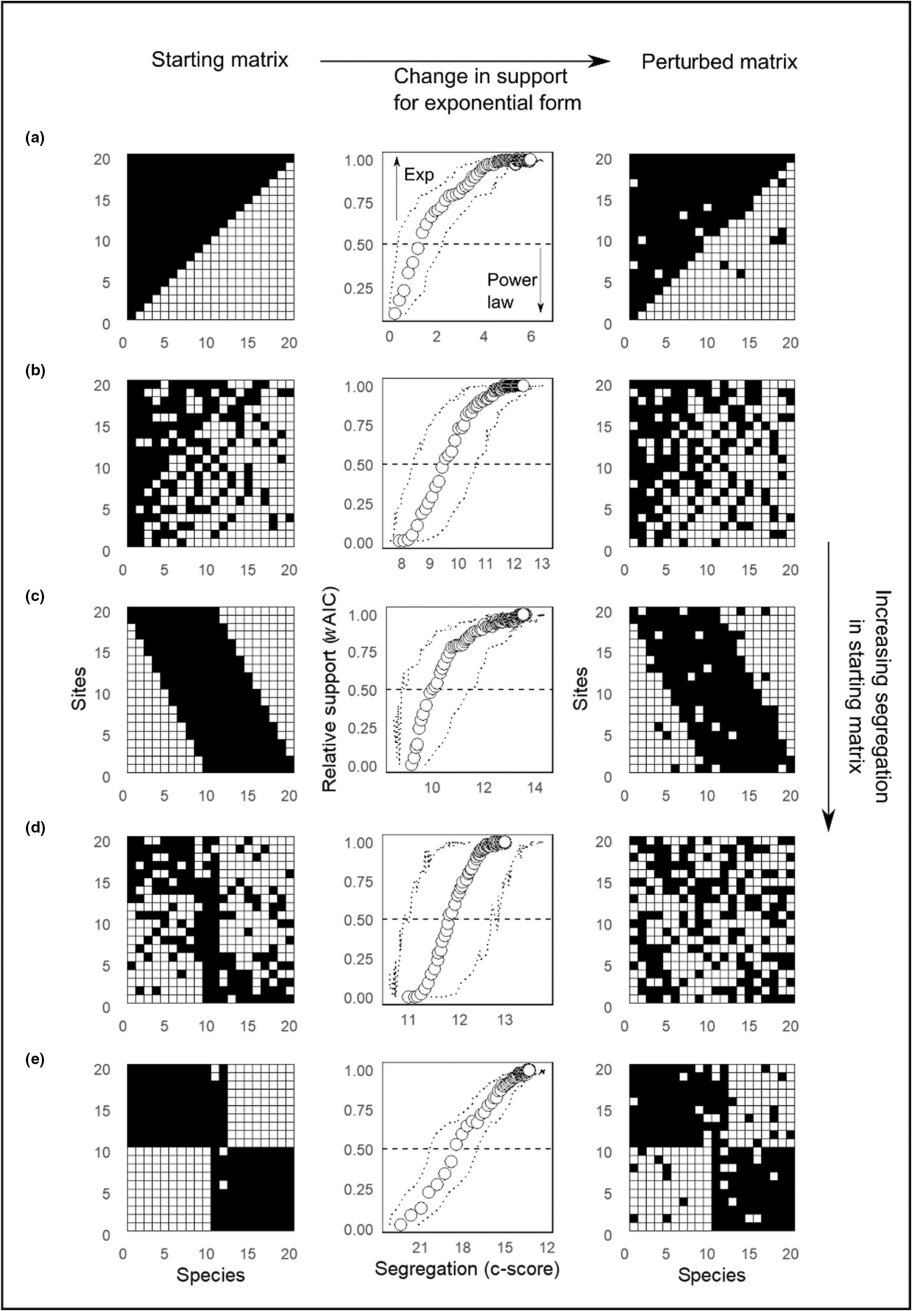
Change in the form of multisite similarity decline with the perturbation of five simulated matrix structures. A value of 1 indicates 100% support for the exponential form. Matrix structures are as follows: (a) nested, (b) continuous community, (c) evenly spaced gradients, (d) ecotone, and (e) turnover compartmented. Starting with the five (a–e) matrix structures on the left (“Starting matrix”), each is sequentially and iteratively changed to a less structured (more random positioning of species) matrix shown in the right‐most panel (“Perturbed matrix”), shifting them toward more random interspecific association. Simulated matrix structures are ordered from the least (a) to the most (e) segregated (quantified using the *C*‐score). In each row, the center panel shows how relative support for the exponential form changes as the matrix structure shown in the left panel is perturbed. Relative support for the exponential compared with the power law as the better of the two models for multisite decline is quantified using the Akaike weight (*w*AIC). See Appendix S2: Figure S2 for results of alternative starting matrix structures.

For constant matrix fill, perturbation increased support for exponential decline for any starting matrix, largely via reducing positive interspecific associations. Of all the starting matrices that were in power law form prior to perturbation, support for the exponential form of decline increased in a broadly linear manner as mean interspecific spatial association (higher *C*‐score) decreased (Figure 3). There were clear differences in the form of decline among the different idealized matrix structures (Figure 3, Appendix S2: Figure S2). Starting matrices with either purely random structures (Appendix S2: Figure S2) or extremes of interspecific segregation (e.g., strict Clementsian communities; Appendix S2: Figure S2) were initially of exponential form. This was unaffected by increasing perturbation of the matrix (*w*AIC remained near 1; Appendix S2). We also found that in simulated matrices, the addition of a single species with a saturated distribution (i.e., observed in every site) can change the starting matrix to power law form (Appendix S2: Figure S2a).

### 3.3 Anticipated differences in community biodiversity properties under exponential decline

Collectively, simulations suggest that the critical factor in distinguishing exponential communities is that their most widespread species are relatively restricted, occurring and co‐occurring in relatively few sites compared with power law communities. From this, we reason that the less cosmopolitan distribution of widespread species within exponential communities should be evident in empirical data as more segregated interspecific spatial associations (e.g., higher C‐scores and more negative interspecific associations). Further, because they contain fewer widespread cosmopolitan species (presumably locally abundant, if not dominant), and a smaller fraction of total species number in each site (i.e., lower alpha, relative to gamma diversity), this should allow larger local (within‐site) subpopulations to establish. This would be evident in empirical data as exponential communities have more aggregated conspecific spatial patterns, consistent with the abundance‐based simulations. Further, any such localized conspecific aggregation in exponential communities should result in less even relative abundance (i.e., more dominance) within sites. Conversely, having fewer numerically dominant cosmopolitan species, we anticipated pooled abundance distributions in exponential communities would be more even, as found in the abundance‐based simulations.

### 3.4 Empirical tests of hypothesized relationships in community biodiversity properties

Of the 80 empirical communities assigned to a form of multisite similarity decline, 27 (34%) supported an exponential form, and the remainder (66%) a power law form (described in Section 2.5.2 and Appendix S5: Metadata). There was no evidence that the form of decline was affected by study extent (*z* = 0.02, *p* = .6), total number of species (*z* = −0.74, *p* = .5), broad taxonomic grouping (invertebrates, vertebrates, plants, and protista; *χ* ^2^ = 1.9, df = 3, *p* = .6), ecological realm (i.e., terrestrial, marine, and aquatic; *χ* ^2^ = 3.8, df = 2, *p* = .2), or level of anthropogenic impact (*χ* ^2^ = 2.7, df = 3, *p* = .4), suggesting limited influence of ecological context on the form of decline. There was marginal evidence that, despite fitting the model to a constant number of orders, communities represented by more sites favored exponential decline (*z* = 1.8, *p* = .07).

**FIGURE 4:**
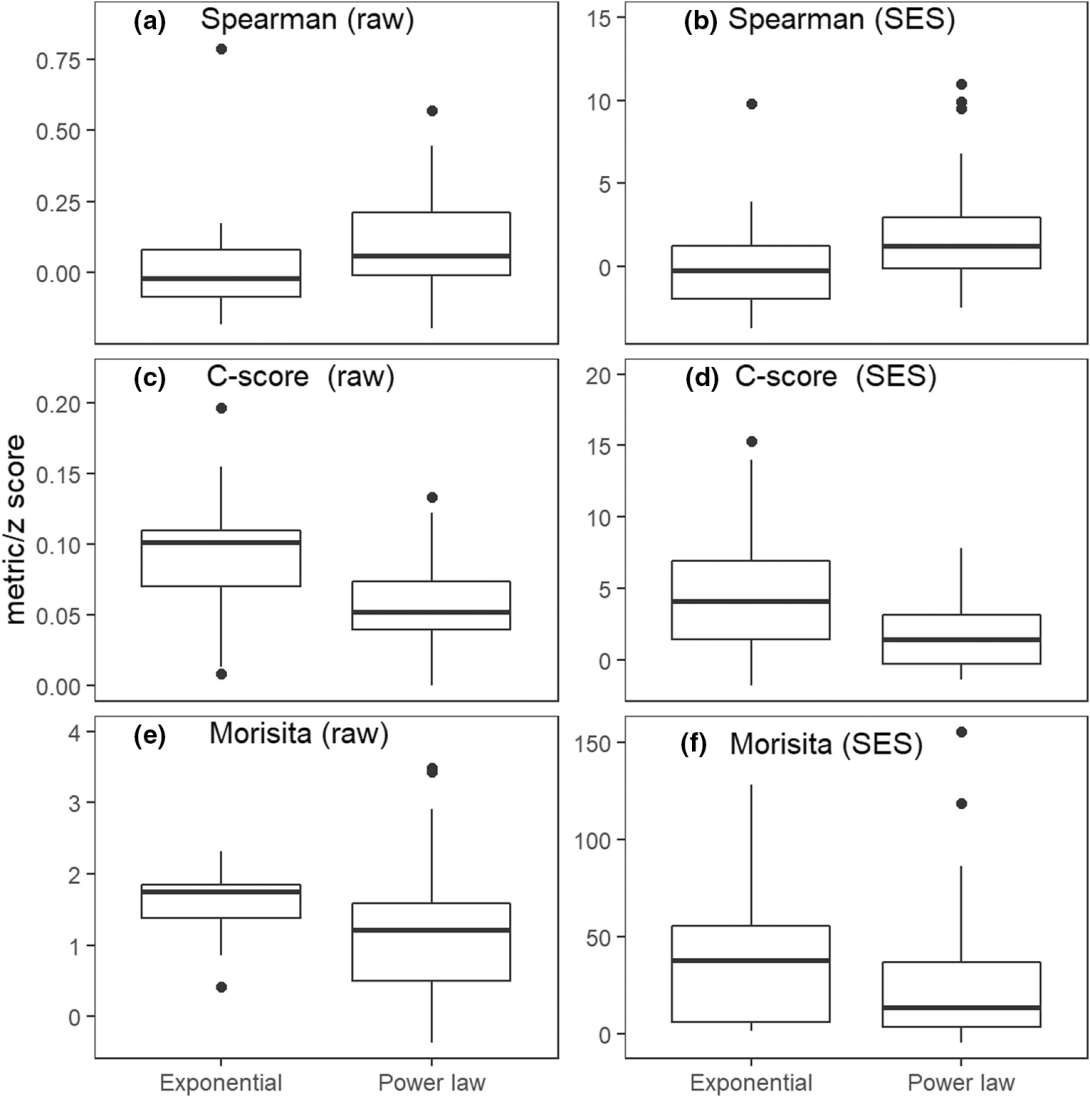
Differences in the distributions of biodiversity properties of communities associated with exponential versus power law multisite similarity decline for the four most abundant species in each community. Top row: interspecific spatial patterns, quantified as the mean Spearman rank correlation. Middle row: interspecific spatial patterns, quantified as the mean *C*‐score. Bottom row: conspecific spatial pattern quantified as mean Morisita index. Raw values are shown in the left column (panels a, c, and e) and standardized effect sizes (SES) in the right (b, d, and f). Communities were assigned to either an exponential or power law form of multisite decline based on the first 10 orders. Standardized effect sizes (SES) were calculated from a reference distribution based on 999 simulated matrices (Section 2). Differences in the distributions in (b) and (d) were significant at the 5% level but those in panel (f) were not (*p* = .1). The upper and lower hinges on each box show the first and third quartiles (i.e., interquartile range), the bold horizontal line shows the median, and vertical lines correspond to 1.5 times the interquartile range.

Interspecific spatial patterns among the four most abundant species in communities following exponential decline showed weaker interspecific association (i.e., were more segregated) than communities with power law decline. This was evident both as weaker correlation in their abundances (median Spearman rank correlation SES: *ρ* = −.10 vs. 1.23, respectively; logistic regression coefficient [SE] = −0.28 [0.13], *z* = −2.22, *p* = .026; Figure 4a,b) and higher *C*‐score (median SES = 3.36 vs. 1.45, respectively; coef. [SE] = 0.25 [0.10], *z* = 2.43, *p* = .015; Figure 4c,d). Despite being numerically greater (Figure 4e,f), conspecific aggregation of the most abundant species did not differ statistically between exponential versus power law forms of decline (median Morisita index SES = 37.7 vs. 14.0, respectively, coef. [SE] = 0.014 [0.008], *z* = 1.61, *p* = .11; Figure 4f). However, for species with greater than median abundance, conspecific spatial patterns were more strongly aggregated in exponential communities (median SES = 30.4 vs. 11.7, respectively, coef. [SE] = 0.019, [0.008], *z* = 2.18, *p* = .030), providing general support for this hypothesis of stronger conspecific aggregation in exponential than power law cases.

Although simulations implied greater evenness in pooled relative abundance distributions would favor exponential communities, there was no empirical evidence of a statistical difference between the two forms of multisite similarity decline (PIE for pooled individuals in all samples; *p* = .50; Appendix S4: Figure S2). In contrast, and consistent with simulations, there was weak statistical evidence that the mean distribution of abundance within sites was less even in exponential than power law form communities (median of sample PIE = 0.66 vs. 0.83, coef. [SE] = −3.83, [2.06], *z* = −1.86, *p* = .063; Appendix S4: Figure S2). Similarly, the ratio of mean sample richness (alpha diversity) to total species richness (gamma diversity) (i.e., matrix fill) was lower in exponential form communities (coef. [SE] = −7.77 [2.68], *z* = −2.90, *p* = .003; Appendix S4: Figure S3a). Additionally, a higher proportion of species were only present at a single site in exponential than power law form communities (coef. [SE] = 5.01 [2.35], *z* = 2.13, *p* = .033; Appendix S4: Figure S3b).

## 4 DISCUSSION

These findings clarify the distinction between the two dominant forms of multisite similarity decline in nature and broadly support our hypothesis of concomitant variation in their respective community biodiversity properties. The most widespread species largely determine the form of decline, and their influence is correlated with characteristic changes in the strength of local‐scale (within site) interspecific associations and conspecific aggregation and relative abundance distributions. From an applied perspective, these results show that a simple stochastic versus niche‐based dichotomy for explaining the two forms are not adequate.

Non‐mutually exclusive conditions that could foster an exponential form include (Table 1) (i) highly differentiated abiotic conditions among sites, or (ii) highly specialized (narrow niche width) taxa, (iii) sampling along successional/disturbance gradients or across ecological range boundaries (e.g., ecotones), and/or (iv) the presence of barriers limiting dispersal of widespread species across the study extent. McGeoch et al. (2019) provide a practical example, where exponential decline was associated with a switch in species dominance across an ecotone in Whittaker's (Whittaker, 1956) Smoky Mountain vegetation gradient. The more differentiated species composition associated with exponential decline – a likely outcome of sampling across range boundaries – is broadly consistent with distance decay of similarity, where exponential decay is similarly associated with sampling across multiple communities and power law decay within a single community (Nekola & McGill, 2014).

### 4.1 Exponential decline is not definitive evidence for stochastic assembly

Exponential decline has to date been assumed to reflect a dominance of stochastic processes in community assembly (Hui & McGeoch, 2014). However, this stochasticity clearly does not apply to the random placement of individuals within sites (Figure 2b); rather, sites must sample species (not individuals) at random from the species pool. The prevalence of such species‐level stochasticity has been estimated from published data at around 10%, most prominently among plants and invertebrates (Deane & He, 2018). Stochastic dispersal of species among sites is a reasonable explanation for the exponential form in at least two empirical communities analyzed here: spider communities of rainforest bromeliads (Gonçalves‐Souza et al., 2010), and vascular plant colonizers of decaying logs in montane forest (Chmura et al., 2016). However, the observed frequency of exponential decline in around one‐quarter to one‐third of empirical data (Hui & McGeoch, 2014; this study) suggests that the non‐stochastic explanations outlined above are equally likely.

It is also noteworthy that our spatially implicit simulation was unable to reproduce exponential decline; that is, the combination of conspecific spatial pattern and evenness in relative abundance (which together determine species‐level occurrence probabilities) only cannot explain exponential decline at a realistic sampling grain. This suggests that the exponential form is not compatible with an assumption that individuals are positioned at random with respect to the individuals of other species (a common assertion in biodiversity theory; McGill, 2010). It implies that any biodiversity theory that assumes random placement of individuals will be unlikely to explain around one‐third of empirical communities, offering a practical example of the importance of interspecific spatial associations as a facet of biodiversity (Keil et al., 2021).

### 4.2 Form of decline in hypothesis generation

Even in the absence of any knowledge of specific mechanisms, understanding how compositional patterns arise from variation in community biodiversity properties improves our ability to interpret patterns and predict change (He & Legendre, 2002; Keil et al., 2021; McGlinn et al., 2019). For example, stronger aggregation in exponential communities should result in local samples that poorly reflect species' relative abundances in the landscape (Green & Plotkin, 2007) as well as lower alpha diversity (He & Legendre, 2002), as we found (Appendix S4: Figure S2). Based on these results, consistent gain of more widespread species (Staude et al., 2022) can be predicted to produce not only higher alpha diversity (Kreft et al., 2006; Lennon et al., 2004) but also more even distributions of abundance, weaker conspecific aggregation, and more positive interspecific spatial associations.

Knowing that the two forms of decline reflect differences in spatial patterns is also useful for testing ecological theory, such as the role of aggregation and segregation on mechanisms of coexistence (Wiegand et al., 2021). For example, the spatial segregation hypothesis (Pacala, 1997) predicts that local patterns of aggregation and segregation from limited dispersal maintain stability and coexistence among species of equal competitive ability. If one sought to test the predictions of this hypothesis, communities following exponential decline would be more likely to provide a suitable study system.

### 4.3 Use of the form of decline in empirical studies

The most common application of multisite similarity decline is to compare the form between taxa, habitats, or conditions of interest within a single ecological context (e.g., Henriksen et al., 2022; Latombe et al., 2019; McGeoch et al., 2019; Reeve et al., 2022). Whenever both forms of decline are found, some questions as to their likely origins are as follows: Does variation in abiotic conditions differ within or among sites? Is there a difference in any barriers to dispersal among sites? Does the number of generalist (broad niche width) taxa differ, or have any such species colonized or expanded their range? Are there differences in the intensity, spatial distribution, or frequency of disturbance? For example, Leihy et al.'s (2018) finding that native and introduced insects in Antarctica follow exponential and power law forms, respectively, suggests that native species are less widely distributed and/or more segregated among islands, while at least some of the introduced insects are found across most islands. This could be due to stochastic factors such as wind‐driven dispersal (Leihy et al., 2018), but could also reflect greater specialization among the native taxa or broader environmental tolerance or resource use among the introduced taxa.

The deeper understanding of the possible influences on the form of multisite similarity decline from this study allows researchers to plan their data collection accordingly. If the intention is to understand multisite similarity decline, data on abiotic variation among sites and dispersal barriers in the landscape will allow a more nuanced interpretation. Also from a methodological perspective, the scale effect of smaller grain size (i.e., decreasing intensity of spatial autocorrelation and association of species with increasing scale; Hui, 2009) increases the likelihood of detecting more and stronger negative associations, and thus the observed form of decline (Figure 2a). This reinforces the importance of appropriate selection of sampling grain for the taxa and processes under investigation (Wiens, 1989). Additionally, for exponential decline, the most widespread species must occupy no more than ~70%–80% of sites (Appendix S2: Figure S2; note that as a direct measure of occupancy, to use this to explain the form of decline would be circular). The information content of species that occur at every site for understanding the drivers of community composition is low; for some ecological questions, it may be appropriate to remove these prior to analysis (McGeoch et al., 2019).

Our results show that multisite similarity decline can provide far more ecological insights than are being asked of it in dichotomous tests between the two dominant forms. Analyzing multisite similarity decline is potentially an information‐rich method of comparing and understanding community structure and ecological processes, yet many questions remain. For example, the exponential form represents an extreme situation, where widespread species contribute little; we might predict that at the opposite extreme, widespread species exert such a pervasive influence that a third model would provide a superior fit to either the exponential or power law form (e.g., sigmoidal symmetric; Hui & McGeoch, 2014; Jenkins, 2011). It seems likely this power law pattern would arise under conditions opposite to those that promote exponential decline (i.e., homogeneous abiotic conditions, many generalist taxa, absence of disturbance, and high levels of dispersal). However, the complexity of underlying forces is oversimplified and inevitably overlooked in dichotomous tests of decline, and its prevalence in nature remains unknown. More generally, we should recognize that multisite similarity decline lies on a continuum, both in terms of its categorical form and the exponent that quantitatively measures the sensitivity of compositional turnover to zeta order, and this too is a clear direction for future development. The power–exponential function (Hui, 2012; Latombe et al., 2019; Lazarina et al., 2019) could offer the basis for organizing all communities according to their multisite similarity decline.

## 5 CONCLUSION

Here, we have demonstrated that the two dominant forms of multisite similarity decline are differentiated mainly by the contribution of widespread species. More cosmopolitan distribution among widespread species in a community increasingly precludes an exponential form and is associated with more positive interspecific association, more even distribution of abundance, and weaker aggregation of conspecific individuals within sites. If sites sample species at random from the regional pool, it will result in exponential decline, but it seems as likely to arise deterministically, where taxa have narrow abiotic niches relative to among‐site variation in abiotic conditions, when sampling across ecological range boundaries (e.g., ecotones), or in the presence of barriers limiting dispersal of widespread species across the study extent. Moving forward, more fruitful use of multisite similarity decline in empirical studies can be achieved by posing a priori hypotheses on the form it will take based on the known or anticipated strength of ecological processes, particularly abiotic sorting and dispersal among sites. Such an approach will also aid researchers in designing studies that are able to differentiate the most important influences on multisite similarity decline.

## AUTHOR CONTRIBUTIONS

**David C. Deane:** Conceptualization (equal); data curation (lead); formal analysis (lead); methodology (lead); visualization (lead); writing – original draft (lead). **Cang Hui:** Conceptualization (supporting); formal analysis (supporting); investigation (supporting); methodology (supporting); visualization (supporting); writing – original draft (supporting). **Melodie McGeoch:** Conceptualization (supporting); formal analysis (supporting); funding acquisition (lead); investigation (supporting); methodology (supporting); project administration (lead); resources (lead); supervision (lead); visualization (supporting); writing – original draft (supporting).

## ACKNOWLEDGEMENTS

The authors thank the handling editor and two anonymous reviewers for insightful comments.

## FUNDING INFORMATION

This research was supported by the Australian Research Council Discovery Project (DP200101680), and CH is supported by the National Research Foundation of South Africa (grants 89967).

## Supporting information


Appendix S1

## Data Availability

Data, metadata, R code, and Appendix S5 Metadata are archived at the Dryad repository https://doi.org/10.5061/dryad.rbnzs7hds.
