# Supplementary material for: Two dominant forms of multisite similarity decline – Their origins and interpretation
Source: Ecol Evol. 2023 Mar 8;13(3):e9859. doi: 10.1002/ece3.9859 (PMC9994616; doi:10.1002/ece3.9859)
Supplement: Supplementary file 1 — Appendix S1 [file ECE3-13-e9859-s001.docx]

# Supporting information for

***Two dominant forms of multisite similarity decline – their origins and interpretation***

# Appendix S1 General form of zeta diversity decline

While for larger orders the exponential form declines faster than the power law, in general the relative behaviour of the two forms of zeta diversity decline depends on their parameter values, which are determined by the dataset. However, to provide some intuition, it is possible to ask how the two forms would appear were they compared across two communities where both alpha diversity (the mean species richness of samples) and total species number were constant. While there is no guarantee that two communities in nature would be found that share these constraints, it illustrates the relative shapes of the two models. In this comparison, the exponential rapidly decays to near zero but correspondingly asymptotes toward total species number more rapidly (Fig. S1).


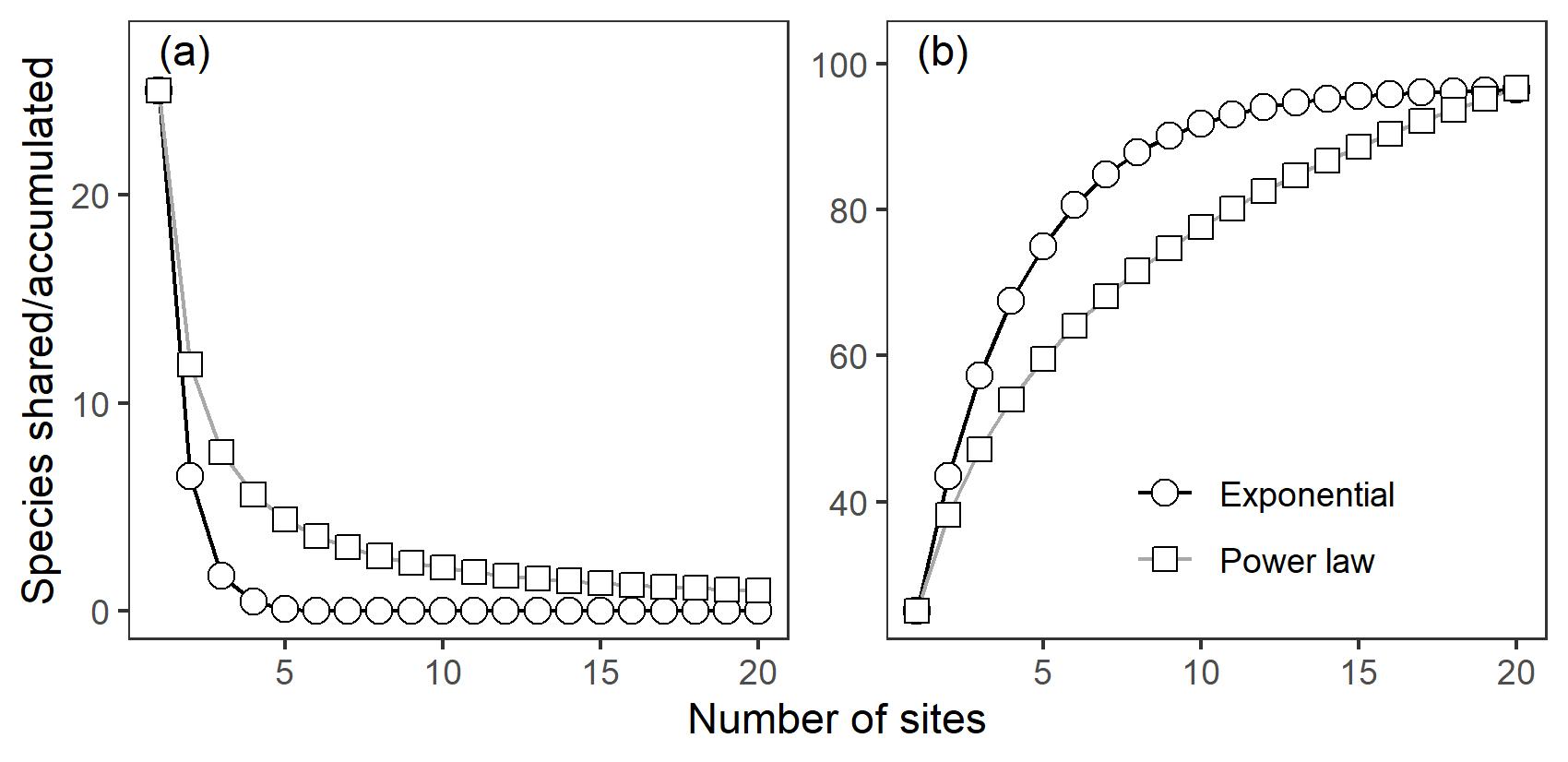


1. Simulation comparing (a) the two forms of decline and (b) species accumulation for two hypothetical datasets with constant alpha (25 species) and gamma (96 species) diversity. The value for alpha diversity and the power law exponent were consistent with the mean value from the Atmar and Patterson matrices analysed in Hui and McGeoch (2014). From the resulting power law decline function $\boldsymbol{\zeta}_{\boldsymbol{i}}\boldsymbol{=25}\boldsymbol{i}^{\boldsymbol{-1.08}}$ a value of 112 for gamma diversity was obtained. This was then used in the exponential decline function, where it corresponds to parameter *a*, and the value for parameter *b* required to obtain an equal alpha value was solved for numerically. The species accumulation curve was calculated from Eq. 1 in Hui and McGeoch.

# Appendix S2 Additional background on simulations

## Conclusions from the abundance-based simulations

Given the abundance-based simulations did not predict the emergence of an exponential form of decline at a reasonable sampling grain for the simulated assemblage, it was assumed to depend on some factor that is not directly incorporated within those simulations. Importantly, these models do not represent the effects of strong environmental gradients or dispersal limitation on compositional turnover (Deane et al., 2022), both of which are likely to increase spatial segregation of species (Chase et al., 2018). Any such segregation would need to apply to all species, particularly the most common and widespread – which would otherwise be expected to be shared across many sites – if exponential decline was to be observed. Abundance-based simulations suggested the most probable (in fact only) way an exponential form of decline would be observed in the absence of environmental gradients and/or dispersal limitation would be where individual samples collected, on average, very few species relative to the overall observed diversity (Fig. 1, main text). This would be most likely when sampling few individuals, corresponding to finer sampling grain (Fig. S1, Fig. 1 main text) and from assemblages with relatively even distributions of abundance and/or high intensity of conspecific aggregation. The latter point is important because it shows that purely stochastic assembly of a community (in the sense that individuals of all species are randomly positioned relative to one another) strongly favours a power law, rather than exponential form of decline.


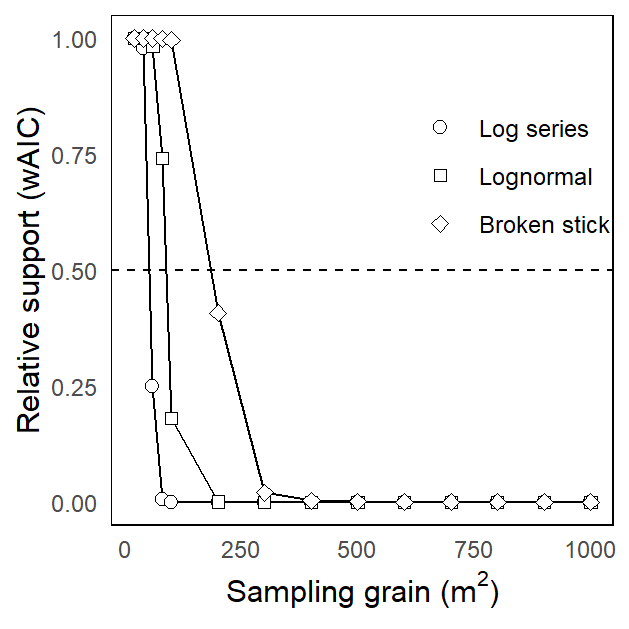


1. Change in support in abundance-based simulations for the exponential form of zeta as a function of sampling grain and different species abundance distribution (see Fig. 1a, main text for detail on sampling grains < 400 m^2^). Relative support for the exponential compared with the power law as the better of the two models for zeta decline was quantified using the Akaike weight (*w*AIC), where a value of 1 indicates 100% weight of support for the exponential form.

## Supporting idealised matrix simulations

In addition to the idealised matrices presented in the main text, a range of other structures were explored. When a strict Clementsian structure is imposed on the matrix (i.e., with no species in common between two assemblages), the resulting segregation strongly favours an exponential form of decline (Fig. 2, main text). However, for a community of the dimension presented (20 sites and 20 species) it required only a single species to be found in every patch for a power law form to be favoured (Fig S2). In this case, power law decline was favoured until the occupancy of the species found at all sites was reduced (on average across 20 simulations) to 16.8 sites. When the number of species found at every site increased to three species, it required more perturbation of the idealised structure to allow for an exponential form to emerge. This corresponded to a situation where no species was found at more than 17.7 sites. This suggests that the exponential form will not typically arise if any species occurs at every site, but multiple widespread species do not necessarily preclude the exponential if none are present at every site.

As expected, when species were assigned to sites at random, support for the exponential form remains near to 1 irrespective of any further perturbation of the matrix (Fig S2c). This affirms that stochastic assembly among patches will result in an exponential form of decline, but clearly it is possible that any deterministic mechanism that segregates species adequately in space can also produce an exponential form of decline. In other words, observing an exponential decline in a group of samples is not necessarily a hallmark of stochastic assembly, although it is a possible explanation.

42
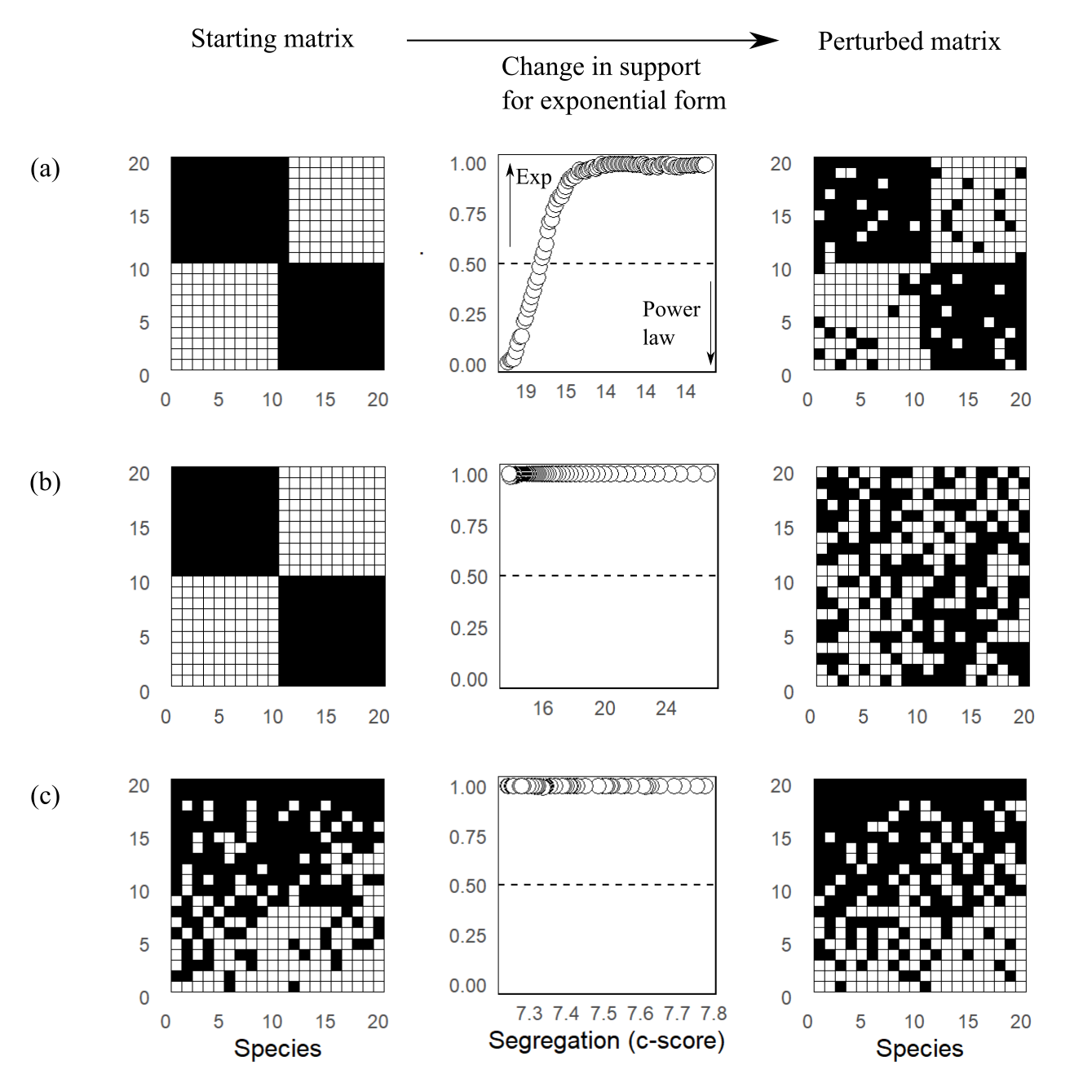


1. Relationship between matrix structure and the form of zeta decline (exponential or power law) from additional community simulations, illustrating sensitivity to widespread species (a-b) and truly random structure (c). (a) Clementsian communities with overlapping range in one species such that it is present at every site ('turnover compartmented'; Ulrich and Gotelli, 2013); (b) ‘true’ Clementsian communities with no shared species (c) random matrix. Each row shows the starting matrix on the left, change in relative support for the exponential form (averaged over 100 simulations) and the final matrix structure after perturbations on the right. Relative support shown on the *y*-axis in the centre panels is quantified using the Akaike weight (*w*AIC), which increases as the strength of support for the exponential form as the better model increases relative to the evidence for the power law. Note that in panels (a) and (b) the C-score is shown in the order of simulations and the maximum score was found in the idealised matrix, but the exponential form was not supported because of the species found in every site.

# Appendix S3 On the classification of datasets as exponential or power law forms of decline

Whenever fitting a model of zeta decline, the researcher must decide on an appropriate number of orders to fit the model. While to some extent this will be informed by the question under investigation, this decision also affects the probability of obtaining one or the other form. Fitting to a high number of orders increases the likelihood of finding an exponential decline (Fig S1), yet researchers should be alert to situations where the increased support for the exponential form when fitting to many orders of zeta simply reflects the better of two poorly fitting models (Fig. S1). For clarity and reproducibility, the choice of approach should be specified by the researcher.


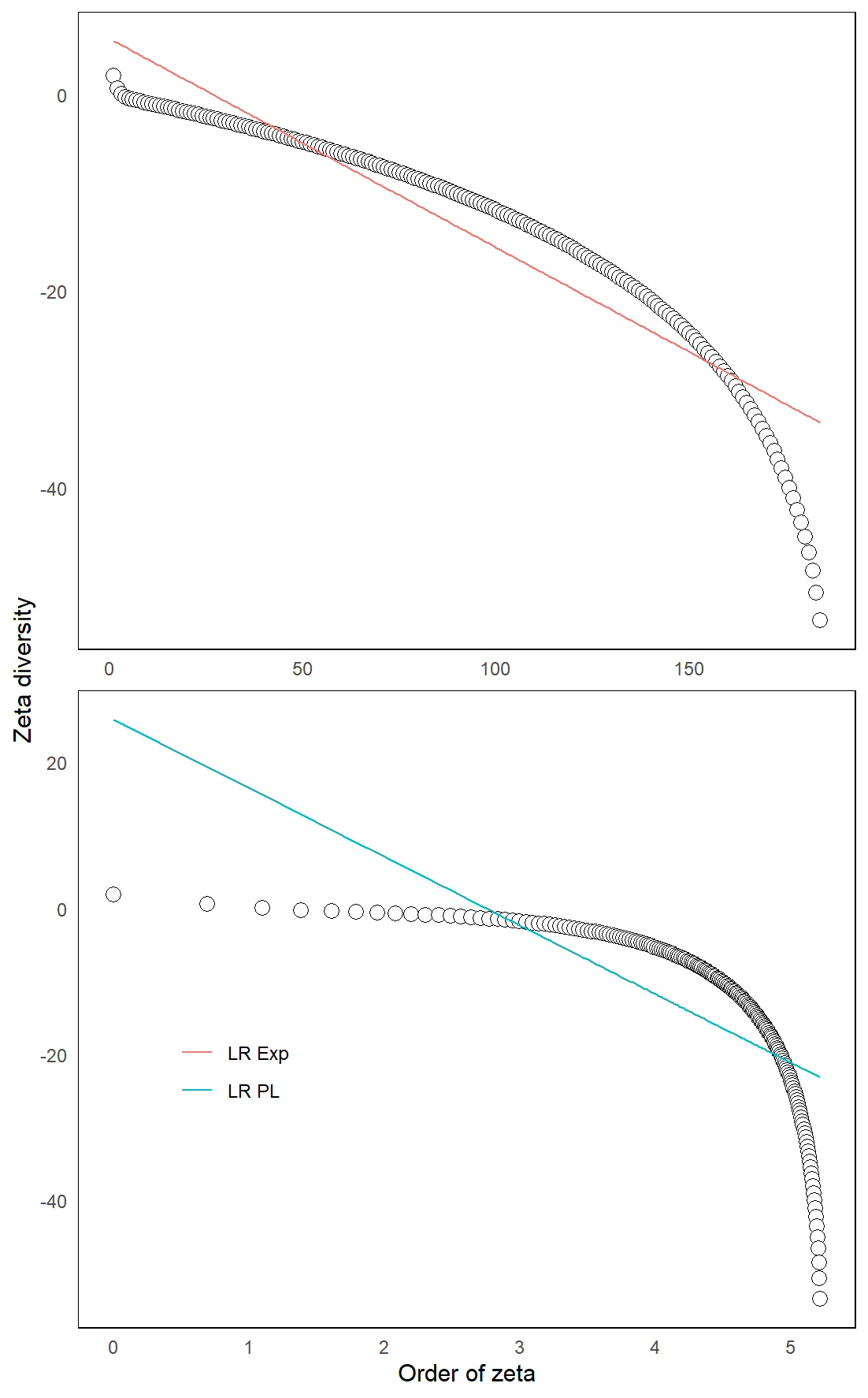


1. Fitting to many orders increases the likelihood of the exponential form being superior in fit to the power law, despite neither model fitting the data well. The use of transformed data was particularly sensitive to this. In this example, linear regression on all orders where the value of zeta exceeded 0 best supported exponential decline (AIC = 1064) over a power law decline (AIC = 1298). However, neither model provides a good fit to the data and the nonlinear model arrives at the opposite conclusion (Fig. S4.2).

To avoid differences relating to the number of sites, we fit to a constant number of orders, here 10, manually verifying each model fit more appropriately described the data (that is, to ensure fitting to higher orders resulted in a poor model fit). Note that using all orders where zeta remained above 1 but fitting to a minimum of 10 sites regardless of zeta value yielded the same grouping of communities. Results also remained qualitatively the same when a minimum of 5 orders was used (see Appendix S4: Figure S1).

## Recommended maximum number of orders of zeta to calculate for assigning form of decline

One way to approach the decision of maximum order is to limit the number of orders used to those where the value of zeta diversity remains above 1 (Latombe et al., 2019). However, whether the numerical value of zeta diversity remains above 1 for a given number of orders is highly sensitive to the occupancy of the most widespread species (Fig. S2) In 40 of the 80 empirical communities analysed, zeta diversity remained above 1 for fewer than 6 orders (20 with less than 3 orders). This can be understood as follows.


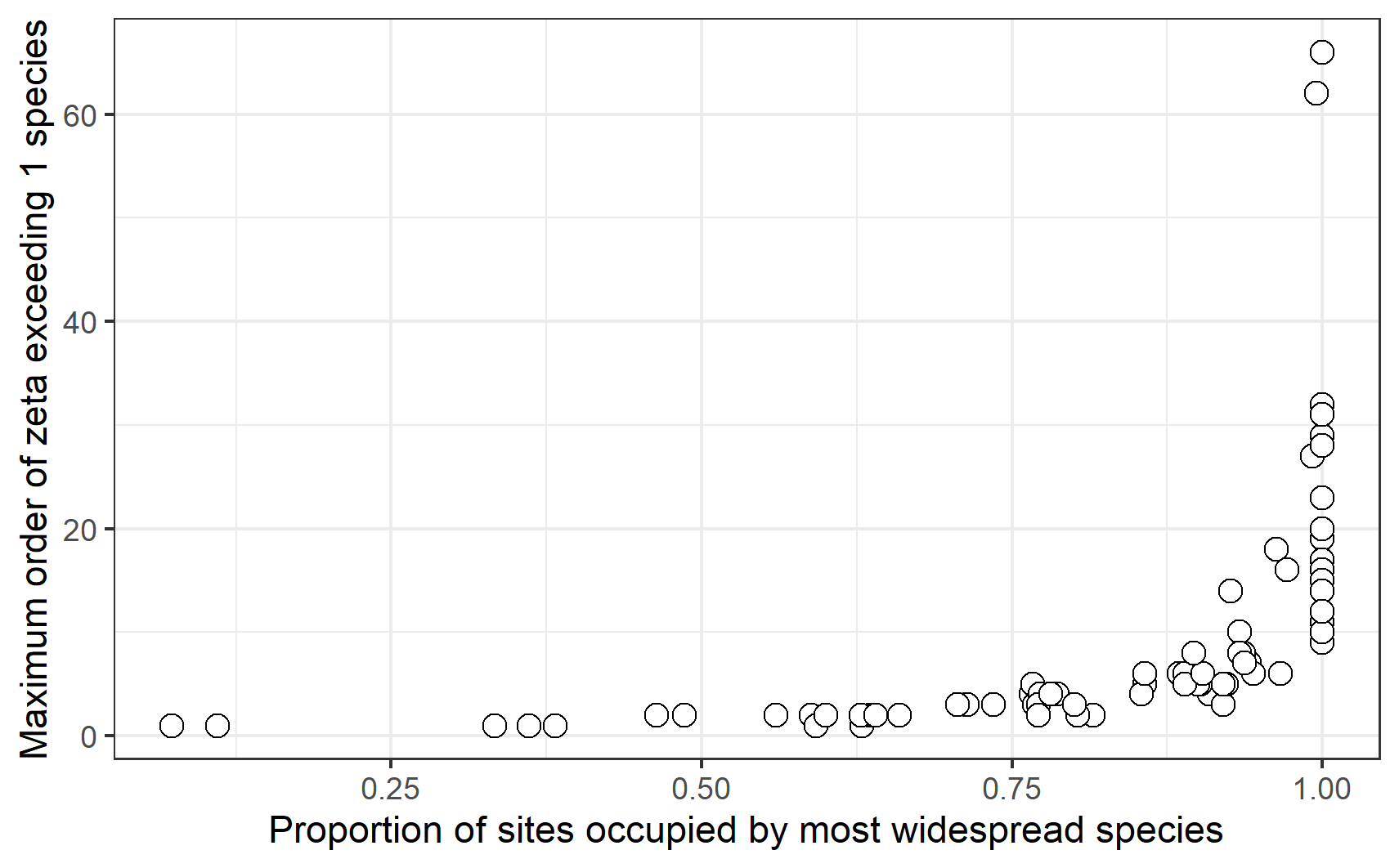


1. The number of orders where zeta diversity remains above 1 depends on the most widespread species. If the most widespread species is found at less than ~75% of sites, the value of zeta falls rapidly falls below 1, typically within 3-4 orders. Fitting zeta to a greater number of orders is ecologically valid but requires careful inspection of the plot to ensure the better performing model describes the data well (see also Table S4.1).

Let *n*_1_ = max(*n_i_*), where n*_i_* is the number of sites occupied by species *i*. Then, the chance to have the most widespread species in a combination of *k* sites is ${\binom{\boldsymbol{n}_{\boldsymbol{1}}}{\boldsymbol{k}}}/{\binom{\boldsymbol{n}}{\boldsymbol{k}}}$, where *n* is the total number of sites. When *k* > *n*_1_, zeta becomes strictly zero, so theoretically the maximum order one can explore should be *n*_1_, the number of sites occupied by the most widespread species. This means the minimum non-negative zeta is ${\binom{\boldsymbol{n}_{\boldsymbol{1}}}{\boldsymbol{n}_{\boldsymbol{1}}}}/{\binom{\boldsymbol{n}}{\boldsymbol{n}_{\boldsymbol{1}}}}\boldsymbol{=}\boldsymbol{1}/{\binom{\boldsymbol{n}}{\boldsymbol{n}_{\boldsymbol{1}}}}\boldsymbol{,}$which approaches ${\boldsymbol{n}_{\boldsymbol{1}}\boldsymbol{!}}/{\boldsymbol{n}^{\boldsymbol{n}_{\boldsymbol{1}}}}$ (Stirling's approximation for *n* approaching infinity and ${\boldsymbol{n}_{\boldsymbol{1}}}/\boldsymbol{n}$ approaching zero).

Let $\boldsymbol{p}_{\boldsymbol{i}}\boldsymbol{=}{\boldsymbol{n}_{\boldsymbol{i}}}/\boldsymbol{n}$, then zeta is approximately $\boldsymbol{\zeta}_{\boldsymbol{k}}\boldsymbol{=}\sum_{\boldsymbol{i=1}}^{\boldsymbol{s}} {\boldsymbol{p}_{\boldsymbol{i}}}^{\boldsymbol{k}_{\boldsymbol{i}}}$ and $\boldsymbol{\zeta}_{\boldsymbol{k}}$ approaches the number of species occupying all sites. If $\boldsymbol{p}_{\boldsymbol{1}}\boldsymbol{=max}\left[ \boldsymbol{p}_{\boldsymbol{i}} \right]\boldsymbol{<1}$, then $\boldsymbol{\zeta}_{\boldsymbol{k}}$ approaches zero, and the rate is roughly $\boldsymbol{\zeta}_{\boldsymbol{k}}\boldsymbol{\sim}\left( \boldsymbol{p}_{\boldsymbol{1}} \right)^{\boldsymbol{k}}$, which is exponential. For zeta diversity to exceed 1 there is at least one species occurring in all sites. So, the recommended range of zeta shouldn't be for zeta >1, instead it is theoretically *n*_1_, the occupancy of the most widespread species. For regression with covariates to explain the variation of zeta, then, say, we require at least 5% non-zero values in calculating zeta, then at least ${\binom{\boldsymbol{n}_{\boldsymbol{1}}}{\boldsymbol{k}}}/{\binom{\boldsymbol{n}}{\boldsymbol{k}}}$, thus $\boldsymbol{k<}{\boldsymbol{ln}\left( \boldsymbol{0.05} \right)}/{\boldsymbol{ln}\left( \boldsymbol{p}_{\boldsymbol{1}} \right)}$. For example, if *p*_1_ = 0.7, then *k* < 8.4.

This leads to the following recommendations for the maximum order of zeta:

If *p*_1_ = 1, then the maximum number of orders should be the total number of sites

If *p*_1_ < 1, then the maximum number of sites, while theoretically *n*_1_, should be limited to *k* < min(*n*_1_, ln(0.05)/ln(*p*_1_)).

However, it is recommended that at least 5 and possibly up to 10 sites are used to ensure the two forms can be reliably distinguished. It is also important to manually inspect the plot to ensure the better fitting model describes the data well.

# Appendix S4 Supplementary results

## Sensitivity to the selection of widespread species

As discussed in the main text, although there was no statistical difference in the distribution of gamma diversity (i.e., total species number) values in datasets following exponential or power law decline, we nonetheless based our comparison of the form of decline on a constant number of species, where the 4 most abundant species were used to calculate raw metrics of interspecific and conspecific spatial patterns. To test sensitivity of the results to this decision, we also used the highest quartile abundances. Results of using this threshold of abundance were qualitatively identical to using four species, with exponential communities having more negative interspecific association than power forms (median SES = 0.38 vs 4.2 respectively, KW χ^2^= 11.2, *df* = 1, *P* < 0.001) higher *C*-score (median SES = 3.4 vs 1.5, χ^2^ = 4.1, df = 1, *P* = 0.04) and more aggregated conspecific spatial pattern, albeit with only marginal statistical significance (median SES = 39.9 vs 15.4, χ^2^ = 3.3, *df* = 1, *P* = 0.07).

## Sensitivity to the selection of maximum orders

As discussed in the main text and Appendix S3, the best supported form of decline is sensitive to the number of orders to which the models are fit. In the main text we present results when the best supported form is based on a comparison of ten sites. Here we show the main conclusions are robust to this decision, presenting the results for a maximum of either 10 sites or the number of orders where zeta diversity remains above 1.


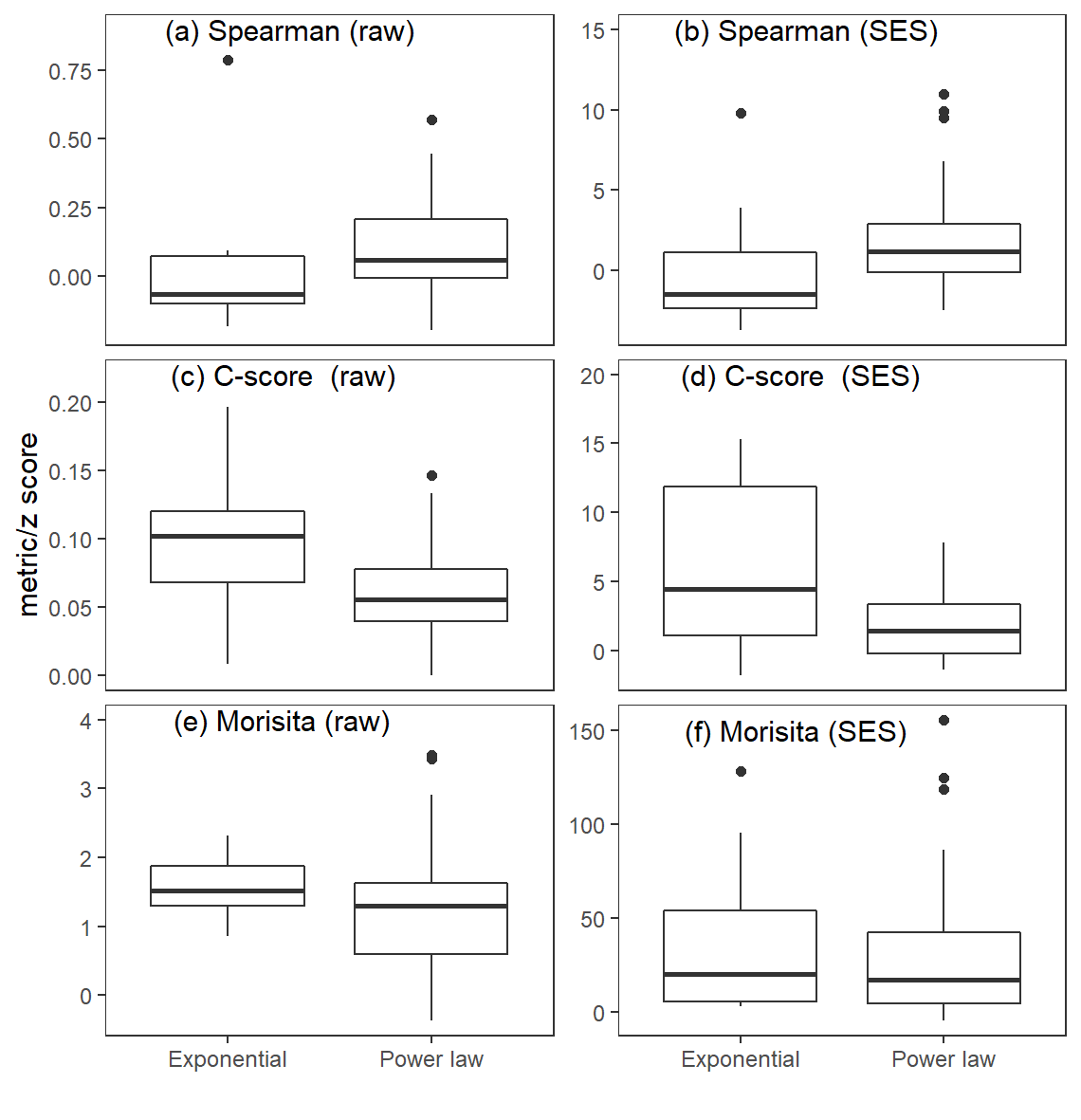


1. Comparison of distribution of community structural metrics between communities following exponential and power law forms of zeta decline based on all orders with a value > 1, with a minimum of five orders (using a minimum of ten orders results in the same grouping as the main text – compare Fig. 3). Left column panels show raw values, and right column panels show the corresponding standardized effect size (*z*-score) calculated from 999 simulated matrices. The upper and lower hinges on each box show the first and third quartiles (i.e., interquartile range), the bold line horizontal line shows the median and vertical lines correspond to 1.5 times the interquartile range.


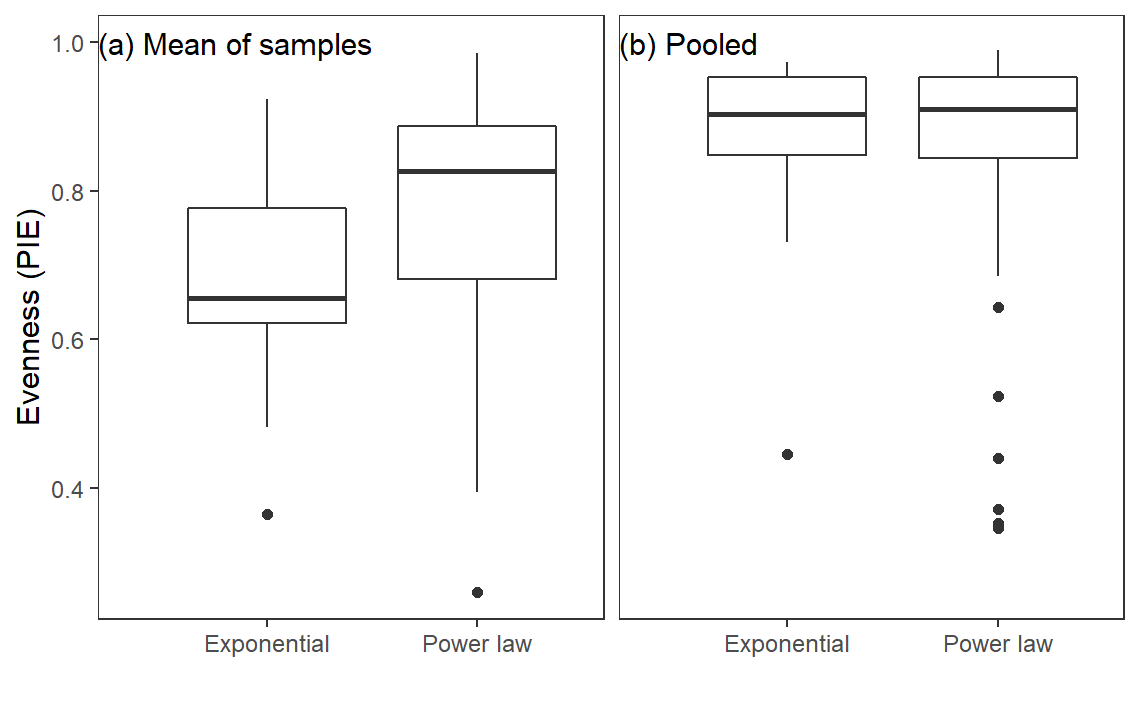


1. Mean sample-level evenness in abundance was lower in exponential than power law decline empirical communities while pooled evenness does not differ. Evenness was quantified as the probability of interspecific encounter (PIE) and panels represent (a) mean PIE of individual samples and (b) the PIE for the community-wide pooled abundance of all species in the data. Upper and lower hinges on each box show the first and third quartiles (i.e., interquartile range), the bold line horizontal line shows the median and vertical lines correspond to 1.5 times the interquartile range.


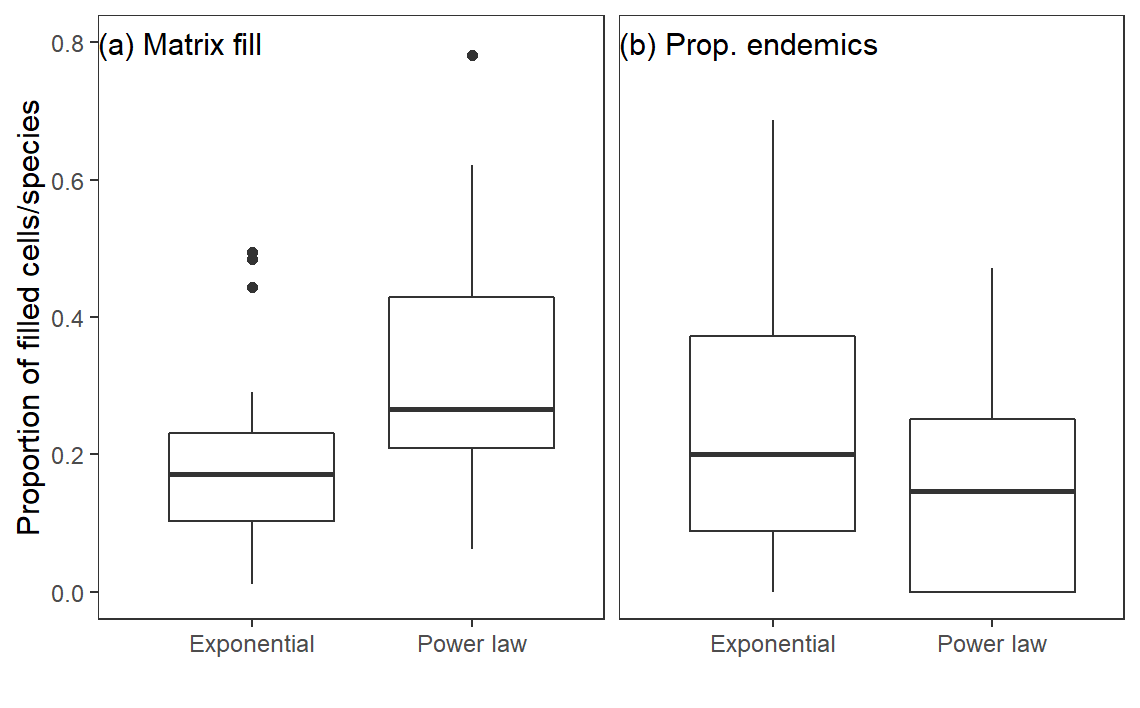


1. Differences in the distribution of matrix fill and proportion of species found in a single patch between exponential and power law forms of zeta decline. Matrix fill is equivalent to the connectance and to the ratio of mean sample richness and total dataset richness (i.e., ${\bar{\boldsymbol{\alpha}}}/\boldsymbol{\gamma}$). The upper and lower hinges on each box show the first and third quartiles (i.e., interquartile range), the bold line horizontal line shows the median and vertical lines correspond to 1.5 times the interquartile range.


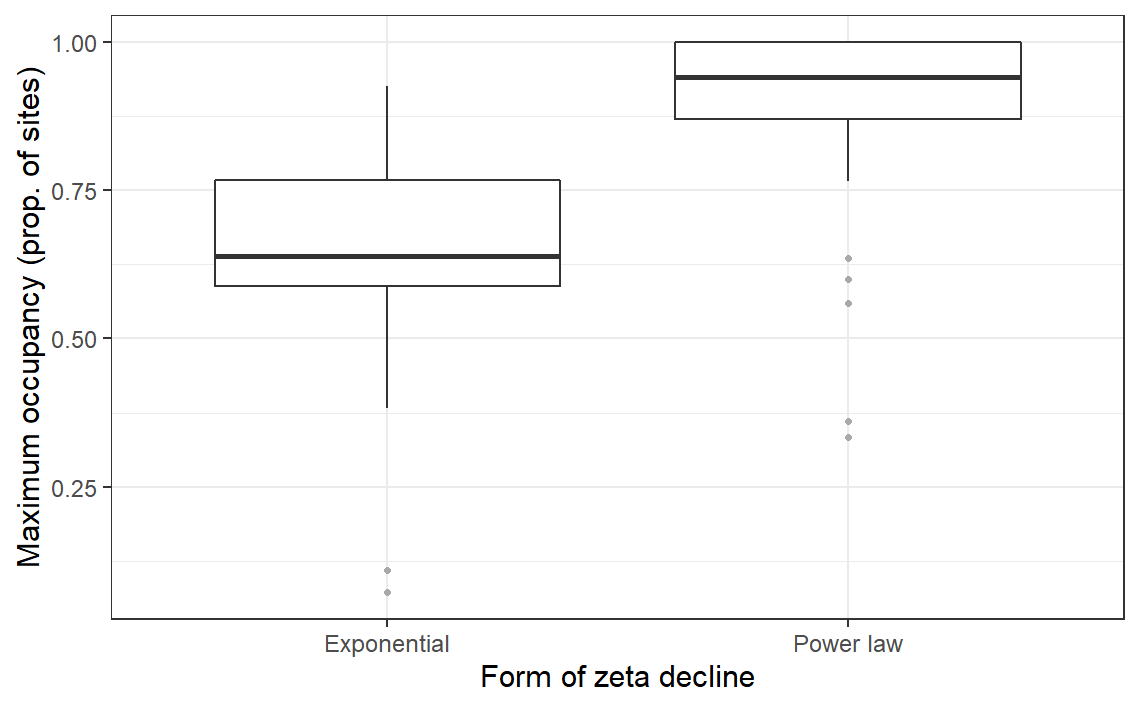


1. The proportion of sites occupied by the most widespread species in communities following an exponential or power law in empirical data. In 35% of power law communities, at least one species was found at every site, but this was never observed for exponential communities. The upper and lower hinges on each box show the first and third quartiles (i.e., interquartile range), the bold line horizontal line shows the median and vertical lines correspond to 1.5 times the interquartile range.

## Correlation among covariates


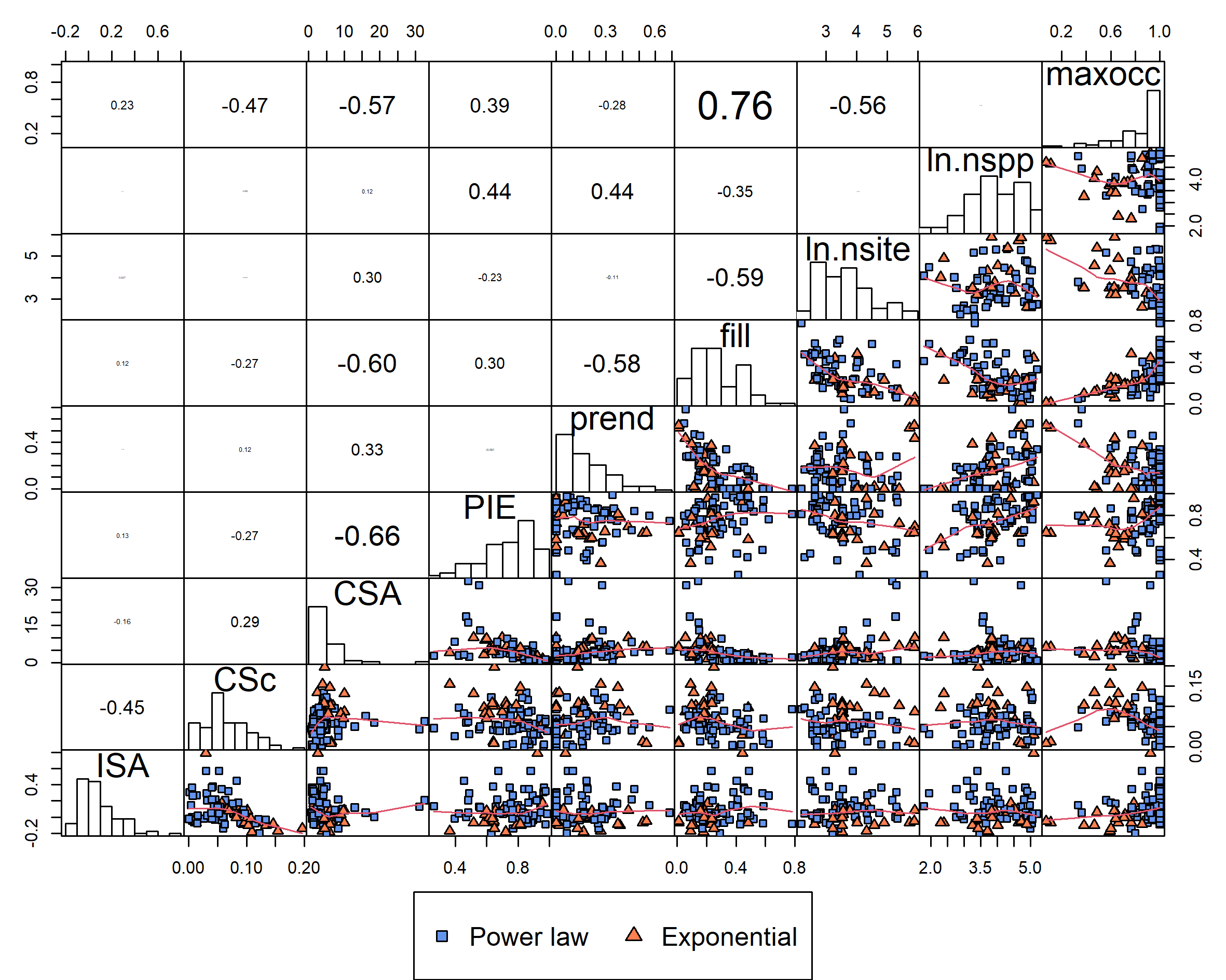


1. Spearman correlation of community properties and assignment to exponential or power law decline for empirical communities (upper triangle plots), distribution of all values (diagonal plots) and scatterplots (lower triangle plots). ln.CSA = natural logarithm of Morisita’s index; ln.nspp = number of species (log transformed); ln.nsite = number of sites (log transformed); prend = proportion of endemic species; PIE = probability of interspecific encounter; CSc = C-score; ISA = interspecific association (Spearman correlation).

# References for Supporting information

CHASE, J. M., MCGILL, B. J., MCGLINN, D. J., MAY, F., BLOWES, S. A., XIAO, X., KNIGHT, T. M., PURSCHKE, O. & GOTELLI, N. J. 2018. Embracing scale-dependence to achieve a deeper understanding of biodiversity and its change across communities. *Ecology Letters,* 21**,** 1737-1751.

DEANE, D. C., XING, D. L., HUI, C., MCGEOCH, M. & HE, F. 2022. A null model for quantifying the geometric effect of habitat subdivision on species diversity. *Global Ecology and Biogeography,* 31**,** 440-453.

HUI, C. & MCGEOCH, M. A. 2014. Zeta diversity as a concept and metric that unifies incidence-based biodiversity patterns. *American Naturalist,* 184**,** 684-694.

LATOMBE, G., ROURA-PASCUA, N. & HUI, C. 2019. Similar compositional turnover but distinct insular environmental and geographical drivers of native and exotic ants in two oceans. *Journal of Biogeography,* 46**,** 2299-2310.

ULRICH, W. & GOTELLI, N. J. 2013. Pattern detection in null model analysis. *Oikos,* 122**,** 2-18.
